# Association between ambient air pollution exposure and pregnancy outcomes in women treated with assisted reproductive technology: an updated systematic review and meta-analysis

**DOI:** 10.1186/s12889-024-19301-3

**Published:** 2025-05-02

**Authors:** Jian-Chao Qiao, Liang-Jie Sun, Pin-Peng Xie, Zhuo-Yan Li, Meng-Yue Zhang, Si-Yu Gui, Xin-Chen Wang, Jian-Kang Yang, Cheng-Yang Hu

**Affiliations:** 1 Department of Clinical Medicine, The Second School of Clinical Medicine, Anhui Medical University, 81 Meishan Road, Hefei, 230032 China; 2 Department of Ophthalmology, The Second Affiliated Hospital of Anhui Medical University, 678 Furong Road, Hefei, 230601 China; 3 Department of Cardiothoracic Surgery, The First Affiliated Hospital of Anhui Medical University, 218 Jixi Road, Hefei, 230022 China; 4 Department of Humanistic Medicine, School of Humanistic Medicine, Anhui Medical University, 81 Meishan Road, Hefei, 230032 China; 5 Department of Epidemiology and Biostatistics, School of Public Health, Anhui Medical University, 81 Meishan Road, Hefei, 230032 China

**Keywords:** Ambient air pollution, Assisted reproductive technology, ART, Clinical pregnancy, Biochemical pregnancy, Live birth

## Abstract

**Background:**

Ambient air pollution has been recognized as a potential threat to reproductive system function. However, studies investigating the relationship between air pollutants and pregnancy outcomes, particularly in the context of assisted reproductive technology (ART), has yielded inconsistent findings.

**Methods:**

This study conducted an updated comprehensive search to identify observational studies published before October 14, 2023, that examined the associations between air pollution exposure and pregnancy outcomes among women undergoing ART. Meta-analysis using random effects models were employed to calculate pooled risk estimates of clinical pregnancy, biochemical pregnancy, and live birth.

**Results:**

A total of 20 studies were included in the systematic review and meta-analysis, with 12 studies included in the quantitative synthesis. The results revealed that exposure to carbon monoxide (CO) (RR = 0.949, 95% CI: 0.900, 0.999; I^2^ = 73%) and nitrogen dioxide (NO_2_) (RR = 0.976, 95% CI: 0.961, 0.992; I^2^ = 10%) during the period from ovarian stimulation to oocyte retrieval was associated with lower clinical pregnancy rates. Similarly, exposure to CO (RR = 0.985, 95% CI: 0.975, 0.996; I^2^ = 0%) and NO_2_ (RR = 0.978, 95% CI: 0.961, 0.996; I^2^ = 27%) during this period reduced biochemical pregnancy rates.

**Conclusions:**

Our study highlights the potential association between air quality and ART outcomes, underscoring the need for improvements in air quality to enhance reproductive success.

**Supplementary Information:**

The online version contains supplementary material available at 10.1186/s12889-024-19301-3.

## Background

Infertility, a significant reproductive health concern, affects approximately 15% of couples of reproductive age globally [1]. Assisted reproductive technology (ART) offers an effective solution for infertile patients seeking to achieve pregnancy. Since 1981, ART—fertility treatments involving the handling of both oocytes and embryos—has been increasingly utilized in the United States to address infertility [2, 3]. In 2016, over 260,000 ART cycles were conducted at US fertility clinics, resulting in nearly 77,000 live births [3]. In vitro fertilization (IVF) is the most prevalent form of ART, with numerous risk factors influencing its reproductive outcomes. In addition to clinical factors, various environmental factors, including heavy metals [4], chemicals [5], and air pollution [6], among others, can significantly influence conception rates in IVF cycles.

Ambient air pollution, recognized as a significant global public health concern, has consistently been associated with a range of adverse health outcomes. Extensive research has underscored its links to various conditions, including cardiovascular diseases, lung cancer, and asthma, among others [7, 8]. Furthermore, exposure to ambient air pollutants has been linked to infertility [9, 10] as well as a range of adverse perinatal outcomes. These include early pregnancy loss [11, 12], small for gestational age (SGA) [13], preterm birth (PTB) [14, 15], and low birth weight (LBW) [16, 17]. Nevertheless, the relationship between air pollution and ART pregnancy outcomes has yielded inconsistent conclusions. A large population-based study utilizing national data in the USA reported a weak positive association between ozone (O_3_) exposure and implantation and live birth rates [18]. In contrast, a study conducted in Korea found no correlation between O_3_ exposure and IVF outcomes [19]. One study showed that exposure to carbon monoxide (CO), fine particulate matter (PM_2.5_), and nitrogen dioxide (NO_2_) from oocyte retrieval to embryo transfer decreased the rate of biochemical and clinical pregnancies in women under 35 years of age [20]. However, another study conducted in China did not reach the same conclusions regarding the specific effects of air pollution on ART outcomes [21]. Meanwhile, Shi et al. 2021 reported that inhalable particulate matter (PM_10_) increased the risk of lower rate of live birth, while sulfur dioxide (SO_2_) was not associated with any outcomes [22]. The presence of contradictory results can be attributed to several factors, including variations in demographic characteristics, pollutant levels, methods used to estimate pollution exposure [23–25], and the specific time windows of exposure considered [26, 27]. The impacts of long-term exposure to the effects of air pollution are likely greater than those of short-term exposure, as the cumulative effect may increase the sensitivity of chronically exposed populations [28, 29]. These divergences among studies highlight the potential influence of these factors on the outcomes observed. Therefore, it is imperative to account for and carefully consider these factors when interpreting and comparing the findings, thereby ensuring a more comprehensive understanding of the relationship under investigation.

Follicle development in humans is an extensive, multi-stage process. Preantral follicles mature into preovulatory follicles over about 85 days, whereas the complete evolution from primordial to preovulatory follicles unfolds across close to a year [30, 31]. IVF treatment typically unfolds in four distinct phases: ovarian stimulation, oocyte retrieval, embryo transfer, and finally, a pregnancy test. During the ovarian stimulation phase, physicians choose from an array of protocols, including long gonadotrophin-releasing hormone (GnRH)-agonist (-a), short GnRH-a, GnRH antagonist (-ant), or other mild stimulation protocols, based on the woman's age, ovarian reserve markers, and BMI. The duration of ovarian stimulation can span from 8 to 14 days, contingent upon the particular stimulation protocol used [32]. Following this, mature oocytes are extracted from the follicles and then evaluated in the lab to assess their quality and maturity. Resulting embryos are cultured in the laboratory until they advance to the blastocyst stage, at which point suitable embryos are chosen for transfer to the uterus. After the mature oocytes are removed from the follicles, the oocytes are examined in the laboratory to assess their quality and maturity. The resulting embryos are cultured in the laboratory until they reach the blastocyst stage, and then the appropriate embryos are selected for transfer to the uterus. Post-embryo transfer, patients are typically administered progesterone supplements to bolster the development of the endometrium and enhance the chances of successful implantation. Roughly 9–11 days after the embryo transfer, patients undergo a blood test to measure their levels of human chorionic gonadotropin (hCG), a hormone produced by the developing embryo. It is apparent, therefore, that the various stages of ART encompass numerous procedures and interventions that can potentially influence the outcome of the treatment. A detailed examination of these separate stages could unveil sensitive intervals, particularly during the preimplantation phase, that are crucial to the success of the treatment. Examining the relationship between air pollution exposure and IVF treatment results across distinct phases of treatment could pinpoint critical windows that influence pregnancy success, offering valuable insights for women in the initial stages of pregnancy to sidestep days with high pollution levels.

A previously published systematic review and meta-analysis investigated the relationship between air pollution exposure and pregnancy outcomes of women undergoing ART [33]. However, their study included only 14 studies, and our analysis would additionally include newer and more comprehensively relevant studies. The participant populations in the studies of Dai et al. [34] and Jin et al. [35] were similar, and Liu et al. [33] overlapped the results of these two studies in their meta-analysis. In addition, we would also add a comprehensive risk of bias assessment, and detailed assessments of the level of evidence for each exposure-outcome combination, thus strengthening the credibility and comprehensiveness of our analyses.

As a result, we conducted an updated comprehensive systematic review and meta-analysis to synthesize the existing evidence regarding the relationships between exposure to six criteria air pollutants (PM_2.5_, PM_10_, SO_2_, NO_2_, CO, and O_3_) during specific periods of the ART cycle, focusing on their association with reproductive results, namely clinical pregnancy, biochemical pregnancy, and live birth outcomes.

## Materials and methods

This study rigorously followed the guidelines outlined in the Preferred Reporting Items for Systematic Review and Meta-Analysis (PRISMA) to ensure methodological transparency and thoroughness (see Appendix 1). The primary goal was to comprehensively assess the associations between air pollution and pregnancy outcomes in women undergoing ART. Although the systematic review protocol was not pre-published, all procedures were meticulously conducted in accordance with the PRISMA guidelines.

### Search strategy

We systematically performed an online literature search for relevant English-language studies in PubMed, Web of Science, and Embase, published up to October 14, 2023. We constructed the research question based on the PECOS statement (Population, Exposure, Comparator, Outcome, and Study design): “Is air pollution exposure associated with pregnancy outcomes [including biochemical pregnancy, clinical pregnancy, and live birth] in women undergoing assisted reproductive technology in observational studies, considering different exposure levels?” We used search terms such as “air pollution”, “ambient pollution”, “particulate matter”, “sulfur dioxide”, “ozone”, “carbon monoxide”, “nitrogen dioxide”, “assisted reproductive technology”, “artificial insemination”, “in vitro fertilization”, “embryo transfer”, “biochemical pregnancy”, “clinical pregnancy”, and “live birth”. Detailed information about the search strategy can be found in Appendix 2. Additionally, we manually searched the reference lists of excluded reviews and eligible literature.

### Study selection

The study implemented a priori eligibility criteria based on the PECOS statement: (P) Studies involving pregnant women undergoing ART with IVF and intracytoplasmic sperm injection (ICSI), the primary modalities of ART [36, 37]; (E) Studies required to examine exposure to any of the six air pollutants, namely, PM_2.5_, PM_10_, CO, NO_2_, SO_2_, and O_3_; (C) Comparative effect estimates (OR/RR/HR), specifically contrasting outcomes among individuals exposed to varying levels of air pollutants; (O) Studies aimed to explore the incidence or prevalence of various pregnancy outcomes, including biochemical pregnancy, clinical pregnancy, and live birth the studies; (S) Only published human epidemiological studies, specifically cohort, case–control (including nested case-controls), or cross-sectional studies, were included. Strict exclusion criteria were applied: (a) studies involving non-pregnant women; (b) reviews, meeting abstracts, letters, or comments; (c) ecological studies, randomized controlled trials, etc.; (d) studies lacking proper effect estimates for meta-analysis.

The collected studies were imported into EndNote 20, a bibliographic management software, to streamline record management and duplicate removal. Two authors (JCQ and PPX) independently screened the titles and abstracts, assessing their suitability for inclusion via full-text review. Disagreements were resolved through a second review and consultation. Persistent disagreements were referred to a third author (CYH) for final adjudication.

To precisely assess the impact of air pollution exposure on women across different stages of the assisted reproductive process, the meta-analysis included studies encompassing at least one of these periods: “from ovarian stimulation to oocyte retrieval”, “from oocyte retrieval to embryo transfer”, “from embryo transfer to hCG test”, or “from ovarian stimulation to hCG test” [33, 38]. Details of the ART methodology is shown in Table. S1 and Appendix 5. This study concentrated on meta-analysis of pregnant women in fresh assisted reproduction cycles.

Studies focusing on earlier exposure periods (i.e., beginning with a more extended period before ART initiation) and those involving women in non-fresh cycles were descriptively analyzed and systematically reviewed. This methodology was aimed at accurately assessing the influence of air pollution on women during specific ART stages, prioritizing pregnant women in fresh assisted reproduction cycles."

### Data extraction

The two authors (JCQ and ZYL) independently extracted data using a predefined template. The following characteristics were recorded for each study: reference (author and year of publication), study locations and periods, study design (sample size), population age, air pollution exposures, exposure assessment methods, outcomes of interest, statistical models, and covariates adjustment (Table 1).

**Table 1:** Main characteristics of studies included in the systematic review and meta-analysis

Reference (author and year of publication)	Study location and period	Study design (sample size)	Database of the population	ART treatments of the population (age)	Outcomes of interest	windows of exposure	Air pollutant assessed	Air pollutant indicator	Exposure assessment method	Average pollutant concentration	Adjustment covariates	Main finding
Boulet et al. 2019 [18]	USA 2010–2012	Cohort study (253,528)	Centers for Disease Control and Prevention’s National ART Surveillance System	IVF with fresh embryos (all ages)	Clinical pregnancy; Live birth	Cycle start to oocyte retrieval; Oocyte retrieval to embryo transfer; embryo transfer + 14 days	PM_2.5_ and O_3_	National Air Monitoring Stations/State and Local Air Monitoring Stations and numerical output from the Models- 3/Community Multiscale Air Quality	Bayesian downscaler models	PM_2.5_ mean (9.2 to 9.5 μg/m^3^); O_3_ mean (38.1 to 38.5 ppb)	Patient age, parity, infertility diagnosis, number of prior ART cycles, number oocytes retrieved, and season and year of cycle start	Clinical pregnancy: RR = 1.01 (1.000–1.02) for O_3_ from oocyte retrieval to embryo transfer; RR = 1.01 (1.000–1.02) for O_3_ from embryo transfer + 14 days Live birth: RR = 1.01 (1.002–1.02) for O_3_ from cycle start to oocyte retrieval; RR = 1.01 (1.004–1.02) for O_3_ from oocyte retrieval to embryo transfer; RR = 1.04 (1.004–1.03) for O_3_ from embryo transfer + 14 days
Choe et al. 2018 [19]	Korea 2006–2014	Cohort study (4,581)	Clinical records from fertility Centre of the Gangnam CHA Hospital	IVF with fresh embryos (20–44)	Biochemical pregnancy; Clinical pregnancy; Live birth	Start of controlled ovarian stimulation to oocyte retrieval; oocyte retrieval to embryo transfer; embryo transfer to hCG test; start of controlled ovarian stimulation to hCG test	PM_10_, NO_2_, CO, SO_2_ and O_3_	40 air quality monitoring sites in Seoul obtained from the National Institute of Environmental Research	A district average was assigned as individual exposures for women living in the same district	PM_10_ mean (47.6 to 50.6 μg/m^3^); NO_2_ mean (33.6 to 34.8 ppb); SO_2_ mean (5.3 to 5.5 ppb); CO (664.2 to 681.5 ppb); O_3_ mean (34.0 to 34.5 ppb)	Women’s age, BMI, number of oocytes retrieved, ICSI, number of embryos transferred, blastocyst transfer, season and year of oocyte retrieval	Clinical pregnancy: HR = 0.93 (0.87–0.99) for NO_2_ and HR = 0.94 (0.89–1.00) for CO from start of controlled ovarian stimulation to oocyte HR = 0.92 (0.85–0.99) for PM_10_, HR = 0.93 (0.86–1.00) for NO_2_ and HR = 0.93 (0.87–1.00) for CO from embryo transfer to hCG test Biochemical pregnancy: HR = 1.17 (1.04–1.33) for PM_10_ and HR = 1.18 (1.03–1.34) for NO_2_ from embryo transfer to hCG test
Dai et al. 2021 [34]	China 2013–2019	Cohort study (6,659)	Reproductive Medicine Management System from the fertility center of the First Affiliated Hospital of Zhengzhou University Clinical	IVF with fresh embryos (20–49)	Clinical pregnancy; Live birth	85 days before oocyte retrieval; Gn start to oocyte retrieval	PM_2.5_, PM_10_, AQI	Daily China National Environmental Monitoring Center	Average daily concentration for PM_2.5_, PM_10_ and AQI	85 days before the oocyte retrieval: PM_2.5_ mean 73.19 μg/m^3^, PM_10_ mean 130.71 μg/m^3^, and AQI mean 116.40 Start of Gn to oocyte retrieval: PM_2.5_ mean 73.55 μg/m^3^, PM_10_ mean 130.62 μg/m^3^, and AQI mean 117.11	Patient characteristics, including maternal age, BMI, number of retrieved oocytes, education, infertility types, fertilization method, and protocols were obtained from the CCRM system	Clinical pregnancy: OR = 0.838 (0.723–0.971) for PM_2.5_ and OR = 0.818 (0.705–0.950) for PM_10_ during 85 days before oocyte retrieval; OR = 0.852 (0.736–0.987) for PM_2.5_ from Gn start to oocyte retrieval Live birth: OR = 0.852 (0.736–0.987) for PM_2.5_ and OR = 0.850 (0.733, 0.986) for PM_10_ 85 days before oocyte retrieval; OR = 0.817 (0.733, 0.986) for PM_2.5_ from Gn start to oocyte retrieval
Gonzalez-Comadran et al. 2021 [39]	Spain 2014–2018	Cohort study (194)	Clinical records from Hospital del Mar de Barcelona, CIRH and Hospital Universitari Quirón-Dexeus	IVF with fresh versus frozen-thawed embryos (Clinical pregnancy: 36.44 ± 0.33, No pregnancy: 37.20 ± 0.25)	Clinical pregnancy	15 days before embryo transfer; 3 days before embryo transfer; the same day of transfer; 7 days after transfer	PM_2.5_, PM_10_ and NO_2_	European Study of Cohorts for Air Pollution Effects	Land use regression modeling following a standardized protocol	NO_2_ mean (36.05 to 38.01 μg/m^3^); NO_X_ mean (62.09 to 65.41 μg/m^3^); PM_2.5_ mean (10.35 to 10.57) μg/m^3^; PM_2.5_ab mean (1.62 to 2.02 1 unitat); PM_10_ mean (21.83 to 22.31 μg/m^3^)	Age, BMI, physical activity, smoking intensity, adherence to Mediterranean diet, socioeconomic status, number and quality of the embryos transferred, and the type of endometrial preparation	Clinical pregnancy: OR = 1.84 (1.00–3.39) for PM_2.5_ three days before the embryo transfer
Iodice et al. 2021 [40]	Italy 2014–2017	Cohort study (2,122)	Clinical records from the Centro Scienze Natalit` a of IRCCS Ospedale San Raffaele, Milan, Italy	IVF and ICSI with fresh or frozen embryos (37.3 ± 4.3)	Clinical pregnancy; Biochemical pregnancy	14 days preceding the oocyte retrieval; on the day of oocyte retrieval	PM_10_	Monitoring stations of the ARPA Lombardy air quality network	ARIA Regional Modelling, a chemical-physical model of air quality	Mean Day 0–7: 34.1μg/m^3^; Mean Day 0–14: 34μg/m^3^	Female age, BMI, AMH levels, progesterone levels at hCG triggering, indications to ART cycles, days of the week	Clinical pregnancy: OR = 1.052 (1.002–1.105) from Day-9 to day 0, OR = 1.054 (1.005–1.106) from Day-10 to day 0, OR = 1.066 (1.014–1.120) from Day-11 to day 0, OR = 1.072 (1.020–1.127) from Day-12 to day 0, OR = 1.050 (0.999–1.104) from Day-13 to day 0
Jin et al. 2022 [35]	China 2015–2020	Cohort study (6,564)	Clinical records from the center for reproductive medicine of the First Affiliated Hospital of Zhengzhou University	IVF with fresh embryos (31.2)	Clinical pregnancy	From 90 days before oocyte retrieval to Gn start; from Gn start to oocyte retrieval; from oocyte retrieval to embryo transfer; from embryo transfer to 35 days after transfer; from 90 days before oocyte retrieval to 35 days after transfer	PM_2.5_, PM_10_, NO_2_, SO_2_, CO and O_3_	13 monitoring stations in Zhengzhou	PM_2.5_, PM_10_, NO_2_, SO_2_, CO: 24-h average values of all monitoring stations O_3_: the maximum 8-h rolling average concentration level of all monitoring stations	PM_2.5_ median (42.73 to 50.39 μg/m^3^), PM_10_ median (94.1 to 111.12 μg/m^3^), NO_2_ median (43.67 to 48,51 μg/m^3^), SO_2_ median (11.31 to 12.93 μg/m^3^), CO median (0.92 to 0.99 mg/m^3^) and O_3_ median (114.83 to 128.63 μg/m^3^)	The female's age, BMI, duration of stimulation, No. of retrieved oocytes, baseline FSH, duration of infertility, endometrial thickness on transfer day, No. of embryo transfer, stage of embryos transfer, and year of transfer	Clinical pregnancy: OR = 0.915 (0.869–0.963) for PM_2.5_, OR = 0.924 (0.870–0.982) for SO_2_, OR = 0.894 (0.846–0.945) for CO, OR = 0.920 (0.873–0.970) for NO_2_ and OR = 1.070 (0.870–0.982) for O_3_ from Gn start to oocyte retrieval; OR = 0.929 (0.876–0.985) for SO_2_ and OR = 1.081 (1.030–1.133) for O_3_ from Gn start to oocyte retrieval; OR = 0.894 (0.850–0.941) for PM_2.5_, OR = 0.912 (0.867–0.960) for PM_10_, OR = 0.866 (0.814–0.922) for SO_2_, OR = 0.849 (0.800–0.900) for CO and OR = 0.866 (0.820–0.914) for NO_2_ and OR = 1.113 (1.060–1.167) for O_3_ from embryo transfer to 35 days after transfer
Legro et al. 2010 [6]	USA 2000–2007	Cohort study (7,403)	Clinical records from three centers: Penn State College of Medicine in Hershey, PA, USA; Shady Grove Fertility in Rockville, MD, USA and Columbia University College of Physicians and Surgeons in New York, NY, USA	IVF (35.0 ± 4.5)	Clinical pregnancy; Live birth	From medication start to oocyte retrieval; from oocyte retrieval to embryo transfer; from embryo transfer to pregnancy test (14 days); from embryo transfer to the date of live birth	PM_2.5_, PM_10_, SO_2_, NO_2_ and O_3_	All ambient criteria air pollutant concentration data recorded at monitors operating in the contiguous USA	An established national-scale, log-normal kriging method were used to spatially estimate daily mean concentrations of criteria pollutants at addresses of subjects	PM_2.5_ mean (14.01 to 14.45 μg/m^3^); PM_10_ mean (23.80 to 24.10 μg/m^3^); SO_2_ mean (0.057 to 0.063 ppm); NO_2_ mean (0.018 to 0.019 ppm); O_3_ mean (0.037 to 0.038 ppm)	Patient’s age, IVF center and the year and season of oocyte retrieval	Live birth: OR = 0.76 (0.66–0.86) for NO_2_ from embryo transfer to pregnancy test; OR = 1.26 (1.10–1.44) for O_3_ from medication start to oocyte retrieval; OR = 0.62 (0.48–0.81) for O_3_ from embryo transfer to live birth; OR = 0.90 (0.82–0.99) for PM_2.5_ during embryo culture
Li et al. 2020 [41]	China 2014–2019	Cohort study (9,941)	Clinical records from the Second Hospital of Hebei Medical University, Shijiazhuang City, China	IVF and ICSI with fresh embryos (20–47)	Clinical Pregnancy; Biochemistry pregnancy	Preantral follicle stage to Gn start (75 days); Gn start to embryo transfer (11 days); Gn start to embryo transfer (4 days); embryo transfer to serum hCG test (14 days); primordial follicle stage to serum hCG test (360 days)	PM_2.5,_ PM_10_, NO_2_, SO_2_, CO, and O_3_	Collected daily at 149 monitoring stations located at Hebei province	Spatiotemporal kriging model based on residential addresses	PM_2.5_ mean 70 ± 62 μg/m^3^; PM_10_, 120 ± 85 μg/m^3^; NO_2_, 37 ± 21 μg/m^3^; SO_2_, 30 ± 29 μg/m^3^; CO, 1.2 ± 1.0 mg/m^3^; O_3_, 103 ± 58 μg/m^3^	Maternal age, Education, BMI, basal FSH, duration of infertility, total dosage of gonadotrophin, fertilization method, number of oocytes, number of embryo transferred, and endometrial thickness	Clinical pregnancy: OR = 0.95 (0.90–0.99) for PM_2.5_, OR = 0.93 (0.89–0.98) for PM_10_, OR = 0.89 (0.85–0.94) for NO_2_, OR = 0.94 (0.90–0.98) for SO_2_ and OR = 0.93 (0.89–0.97) for CO from preantral follicle stage to Gn starts; OR = 0.93 (0.88–0.97) for NO_2_ and OR = 0.96 (0.93–0.99) for SO_2_ from Gn start to embryo transfer; OR = 1.08 (1.02–1.14) for O_3_ from embryo transfer to serum hCG test; OR = 0.93 (0.88–0.98) for NO_2_ from primordial follicle stage to serum hCG test
Liu et al. 2022 [26]	China, 2014–2018	Cohort study (8,628)	The assisted reproductive electronic medical record system database of the Reproductive Medicine Center of The Third Affiliated Hospital of Zhengzhou University	IVF with fresh embryos (31.11 ± 4.96)	Clinical pregnancy; Biochemical pregnancy; Live birth	Gn injection to oocyte retrieval; oocyte retrieval to embryo transfer; 1 day after embryo transfer to embryo transfer + 14 days and Gn injection to embryo transfer + 14 days	CO, NO_2_, O_3_, PM_10_, PM_2.5_ and SO_2_	Real-time urban air quality data from the Ministry of Ecology and Environment of the People's Republic of China	Average daily concentrations retrieved from the monitoring station closest to the address of subject or to the study centre	CO median 1.22 mg/ m^3^; NO_2_ median 49 µg/m^3^; O_3_ median 105 µg/m^3^; PM_10_ median 124 µg/m^3^; PM_2.5_ median 61 µg/m^3^ and SO_2_ median 20 µg/m^3^	Patient age, BMI, type of infertility, duration of infertility, infertility diagnosis, year cycle started, number of oocytes retrieved, fertilization method, number of embryos transferred, stage and quality of transferred embryos and endometrial thickness	Clinical pregnancy: OR = 0.81 (0.71–0.92) for PM_10_; OR = 0.82 (0.73–0.93) for SO_2_ 1 day after embryo transfer to embryo transfer + 14 days; OR = 0.87 (0.76–1.00) for PM_10_ from gonadotrophin injection to embryo transfer + 14 days Biochemical pregnancy: OR = 1.55 (1.09–2.19) for PM_10_ from gonadotrophin injection to oocyte retrieval Live birth: OR = 0.88 (0.77–0.99) for PM_10_ 1 day after embryo transfer to embryo transfer + 14 days
Perin et al. 2010 [42]	Brazil 1997–2006	Cohort study (348)	IVF database collected from CEERH, Specialized Center for Human Reproduction	IVF with fresh embryos (19–45)	Live birth	Follicular phase	PM_10_	São Paulo State Environmental Protection Agency	24-h arithmetical average of PM_10_ across all monitoring stations	Q1: ≤ 30.48 µg/m^3^, Q2: 30.49–42.00 µg/m^3^, Q3: 42.01–56.72 µg/m^3^, and Q4: > 56.72 µg/m^3^	The year of IVF treatment and patient’s age	Clinical Pregnancy: OR = 5.05 (1.04–24.51) for PM_10_
Qiu et al. 2019 [21]	China 2014–2018	Cohort study (1,455)	Clinical data were extracted from the patient database used in the fertility center of Shengjing hospital	IVF and ICSI with fresh embryos (33.3 ± 4.1)	Clinical pregnancy	85 days before the oocyte retrieval to start of Gn; start of Gn to oocyte retrieval; oocyte retrieval to embryo transfer; embryo transfer to hCG test; start of Gn to hCG test	PM_10_, PM_2.5_, NO_2_, CO, SO_2_ and O_3_	11 air quality monitoring stations in Shenyang obtained from Shenyang Environmental Protection Bureau of China	Each individual air pollution exposure was represented by the data from the nearest monitor station	PM_2.5_ median (48.25 to 55.48 µg/m^3^); PM_10_ median (91.75 to 102.29 µg/m^3^); SO_2_ median (27.20 to 36.55 µg/m^3^); CO median (0.90 to 0.94 µg/m^3^); NO_2_ median (41.75 to 43.16 µg/m^3^); O_3_ (100.04 to 106.00 µg/m^3^)	Maternal age, BMI, education, year of oocyte retrieval, number of retrieved oocytes, number of transferred embryos, fertilization method and stage of transferred embryos	Clinical pregnancy: OR = 0.87 (0.81–0.98) for O_3_ from Gn starting to oocyte retrieval; OR = 0.86 (0.78–0.95) for O_3_ one day before oocyte retrieval; OR = 1.12 (1.01–1.23) for CO two days before oocyte retrieval
Quraishi et al. 2019 [43]	USA 2012–2013	Cohort study (19,003)	Medical records from a network of private fertility clinics across the United States	IVF with fresh embryos (34.9 ± 4.6)	Clinical pregnancy; Live birth	Period before the cycle to ovarian stimulation	PM_2.5_, PM_10_, and NO_2_	Regulatory monitoring data from the Environmental Protection Agency Air Quality System and Interagency Monitoring of Protected Visual Environments networks	A national spatial model incorporating land-use regression and universal kriging	PM_2.5_ mean 8.7 ± 1.4 µg/m^3^, PM_10_ mean 14.9 ± 3.8µg/m^3^, and NO_2_ mean 9.0 ± 4.7 ppb	Age, BMI, race, and NSES, clinic indicators for Seattle, SF, LA, Baltimore/Chesterbrook, and Rockville	Live birth: RR = 0.96 (0.90–1.02) for PM_2.5_, RR = 0.98 (0.94–1.02) for PM_10_, RR = 0.96 (0.91–1.00) for NO_2_ before IVF start Positive hCG test: RR = 0.98 (0.9–1.02) for PM_2.5_, RR = 0.99 (0.95–1.01) for PM_10_, RR = 0.99 (0.95–1.02) for NO_2_ before IVF start
Shi et al. 2021 [22]	China 2016–2019	Cohort study (2,766)	Clinical records from the Centre for Assisted Reproduction of Shanghai First Maternity and Infant Hospital	IVF and ICSI with fresh or frozen embryos (32.7 ± 3.9)	Biochemical pregnancy; Live birth	From three months before oocyte retrieval to serum hCG test; from serum hCG test to live birth outcome; from three months before oocyte retrieval to live birth outcome	PM_2.5_, PM_10_, NO_2_, SO_2_, CO and O_3_	The concentrations of six criteria air pollutants were obtained from 16 air monitoring stations from the Shanghai environmental monitoring center	Available data from the nearest monitoring station were applied to estimate the concentrations of air pollutants for each participant	PM_2.5_ (34.2 to 36.1 µg/m^3^), PM_10_ (50.4 to 51.4 µg/m^3^), NO_2_ (31.0 to 31.5 µg/m^3^), SO_2_ (8.8 to 9.2 µg/m^3^), CO (0.68 to 0.69 mg/m^3^), O_3_ (93.6 to 110.3 µg/m^3^)	Female age, BMI, educational level, employment status, residential address, duration of infertility, causes of infertility, controlled ovarian stimulation protocols, duration of stimulation, total Gn dose, progesterone levels on trigger day, number of retrieved oocytes, fertilization method, endometrial thickness on the day of embryo transfer, year of embryo transfer, season of embryo transfer, type of embryo transfer, and number of embryo(s) transferred	Biochemical pregnancy: OR = 0.86 (0.75–0.99) for NO_2_ from three months before oocyte retrieval to serum hCG test Live birth: OR = 0.88 (0.79–0.99) for PM_10_ from serum hCG test to live birth outcome; OR = 0.88 (0.79–0.99) for PM_10_ from three months before oocyte retrieval to live birth outcome
Tartaglia et al. 2022 [44]	France 2013–2019	Cohort study (10,763)	The Reproductive Biology Department of Bordeaux University Hospital localised in Bordeaux, France and the Jean Villar Fertility Center localised in Bruges, France	IVF and ICSI with fresh or frozen embryos (Clinical pregnancy: 36.0 ± 5.7 No clinical pregnancy: 37.5 ± 6.2)	Clinical pregnancy	From oocyte retrieval to embryo transfer	PM_10_, PM_2.5_, NO_2_ and O_3_, BC	Air quality monitoring stations provided by ATMO Nouvelle Aquitaine	Pollution levels representative of the air quality of a large geographical area, which is the closest pollution monitoring station and is a good representation of the ambient pollution to which fertility centers are exposed	PM_2.5_ mean 56.5 ± 32.2 µg/m^3^; PM_10_ mean 36.4 ± 27.4 µg/m^3^; BC mean 4.3 ± 3.6 µg/m^3^; NO_2_ mean 56.2 ± 34.1 µg/m^3^; O_3_ mean 148 ± 83.1 µg/m^3^	The season of IVF procedure, the ovarian reserve, woman’s age, BMI, smoking status, the oocyte fertilization method (IVF, ICSI) and site of residence, the number of transferred embryos	Clinical pregnancy: OR = 0.92 (0.86–0.98) for O_3_ during gametes and embryos culture
Wan et al. 2022 [45]	China 2015–2018	Cohort study (3,698)	Clinical records from fertility center of Ruijin hospital affiliated to Shanghai Jiaotong university	IVF and ICSI with frozen embryos (No pregnancy: 33.93 ± 5.00; Clinical pregnancy: 33.64 ± 5.00)	Clinical pregnancy	30 days before embryo transfer	PM_2.5_, PM_10_, SO_2_, CO, NO_2_, and O_3_	Nearest monitor station according to each patient’s address	Each individual air pollution exposure was represented by the data from the nearest monitor station	PM_2.5_ mean 41.07 ± 14.89 μg/m^3^; PM_10_ mean 57.33 ± 15.63 μg/m^3^ SO_2_ mean 12.50 ± 4.54 μg/m^3^ CO mean 0.76 ± 0.16 mg/m^3^ NO_2_ mean 42.49 ± 12.14 μg/m^3^ O_3_ mean 96.83 ± 32.09 μg/m^3^	Maternal age, antral follicle count (AFC), AMH, fertilization method, duration of current infertility, infertility types, embryos scores, transfer times	Clinical pregnancy: OR = 0.906 (0.816–0.989) for NO_2_ and OR = 0.931 (0.881–0.995) for O_3_ 30 days before embryo transfer
Wang et al. 2019 [46]	China, 2013–2016	Cohort study; (11,148)	Cllinical records form the affiliated Chenggong Hospital of Xiamen University	IVF and ICSI with fresh embryos (31.5 ± 4.48)	Clinical pregnancy; Live birth	From oocyte retrieval to embryo transfer/cryopreservation	PM_2.5_, PM_10_, SO_2_, NO_2_, CO and O_3_	Three fixed air quality monitoring stations in the city	Estimation of pollutant concentrations at the IVF clinical site was based on an inverse distance weighting interpolation modeling method	PM_2.5_ median 30.26 μg/m^3^; PM_10_ median 51.99 μg/m^3^; SO_2_ median 12.86 μg/m^3^; CO median 0.66 μg/m^3^; NO_2_ median 30.79 μg/m^3^; O_3_ median 82.6 μg/m^3^	Maternal age, BMI, order of embryo transfer, primary infertility, duration of infertility, diagnosis of tubal problem, polycystic ovary syndrome and endometriosis, basal antral follicle count, starting dose of gonadotrophin, type of GnRH analog, endometrial thickness and pattern, oocyte yield, number of embryos transferred, stage of embryos transferred, presence of top-quality embryos transferred and distance from catheter tip to fundal, type of endometrial preparation and indicators for FET	Live birth: OR = 0.63 (0.53–0.74) for SO_2_ and OR = 0.69 (0.58–0.82) for O_3_ in frozen-thawed embryo transfer cycles Clinical pregnancy: OR = 0.88 (0.78–0.99) for SO_2_ in fresh embryo transfer cycles; OR = 0.64 (0.54–0.75) for PM_2.5_, OR = 0.82 (0.69–0.97) for PM_10_, OR = 0.80 (0.68–0.94) for CO and OR = 0.66 (0.56–0.78) for O_3_ in frozen-thawed embryo transfer cycles
Wang et al. 2023 [47]	China 2015–2018	Cohort study (2,431)	Jiangsu Birth Cohort Study, an ongoing prospective cohort study on women who received ART treatments in the Women's Hospital of Nanjing Medical University and the Suzhou Affiliated Hospital of Nanjing Medical University	IVF and ICSI with fresh or frozen embryos (31.3 ± 4.3)	Biochemical pregnancy; Clinical pregnancy; Live birth	Preantral follicle phase; oocyte growth phase; oocyte selection phase; oocyte maturation phase; the duration from oocyte retrieval to embryo transfer; the duration from embryo transfer to serum hCG test; the duration from hCG test to 30 days after embryo transfer	PM_2.5_	Ground hourly PM_2.5_ concentration measured by a local monitoring station of the National Air Quality Monitoring Network from the China National Environmental Monitoring Center	Exposure assessment method of aerosol optical depth was used to predict the long-term trends of ground-level PM_2.5_ at 1 km spatial revolution	PM_2.5_ mean 45.4 μg/m^3^	Maternal age, BMI, parity, center, maternal education, basal FSH, duration of infertility, infertility type, stimulation protocol, number of transferred embryos, fertilization method and stage of transferred embryo	Clinical pregnancy: RR = 0.98 (0.96–1.00) for PM_2.5_ during oocyte selection phase (20–11 days before oocyte retrieval) Biochemical pregnancy: RR = 1.06 (1.00–1.13) for PM_2.5_ in the duration from hCG test to 30 days after embryo transfer
Wu et al. 2021 [48]	China 2014–2018	Cohort study (20,835)	Clinical data extracted from the database of five reproductive centers in four provincials in Northern China	IVF and ICSI with fresh or frozen embryos (32.37 ± 4.39)	Biochemical pregnancy; Clinical pregnancy; Live birth	85 days prior to oocyte retrieval to oocyte retrieval; Gn start to oocyte retrieval; oocyte retrieval to embryo transfer in fresh embryo transfer cycles; 30 days prior to frozen embryo transfer to embryo transfer in FET cycles; embryo transfer to serum hCG test; 85 days prior to oocyte retrieval to hCG test in fresh embryo transfer cycles; 30 days before frozen embryo transfer to hCG test in FET cycles	PM_2.5_, PM_10_, O_3_, NO_2_, CO and SO_2_	The air monitoring station nearest to the residential site obtained from the China National Environmental Monitoring Centre	Average concentrations of these six air pollutants using data from the nearest monitoring station as approximate individual exposure	PM_2.5_ median (73.00 to 82.31 μg/m^3^); PM_10_ median (115.29 to 119.56 μg/m^3^); O_3_ median (44.87 to 53.39 μg/m^3^); SO_2_ median (19.50 to 20.91 μg/m^3^); NO_2_ median (11.50 to 14.47 μg/m^3^); CO median (0.93 to 1.04 mg/m^3^)	Female age, BMI, smoking status, infertility type, infertility cause, duration of infertility, ovarian hyperstimulation protocol, the year and season of treatment, and residential city, the endometrium preparation regimen and indicators of FET	Biochemical pregnancy: OR = 0.889 (0.827–0.956) for O_3_, OR = 0.904 (0.846 to 0.966) for CO from 85 days prior to oocyte retrieval to oocyte retrieval; OR = 0.895 (0.835, 0.960) for O_3_, OR = 0.907 (0.839 to 0.981) for NO_2_, OR = 0.921 (0.864 to 0.981) for CO from Gn start to oocyte retrieval; OR = 0.920 (0.861 to 0.983) for O_3_, 0.924 (0.857 to 0.995) for NO_2_, OR = 0.936 (0.879 to 0.996) for CO from oocyte retrieval to embryo transfer in fresh embryo transfer cycles; OR = 0.904 (0.846 to 0.966) for CO from embryo transfer to serum hCG test; OR = 0.895 (0.833 to 0.963) for O_3_, OR = 0.901 (0.845 to 0.964) for CO from 85 days prior to oocyte retrieval to hCG test in fresh embryo transfer
Zeng et al. 2020 [20]	China, 2014–2019	Cohort study (1,139)	Clinical records from Reproductive Center of West China Second University Hospital, Sichuan University	IVF and ICSI with fresh embryos (33.50 ± 4.38)	Biochemical pregnancy; Clinical pregnancy	From Gn start to oocyte retrieval; from oocyte retrieval to embryo transfer; from embryo transfer to serum HCG test; from embryo transfer to ultrasound sound test for pregnancy	PM_2.5_, PM_10_, SO_2_, NO_2_, CO and O_3_	The concentrations were obtained from the China National Environmental Monitoring Centre	For each district, we calculated average ambient air pollutant concentration for aforementioned criteria pollutants	PM_2.5_ mean 58.5 ± 43.52 μg/m^3^, PM_10_ mean 95.51 ± 64.81 μg/m^3^, NO_2_ mean 47.77 ± 21.75 μg/m^3^, SO_2_ mean 13.88 ± 8.26 μg/m^3^, CO mean 0.97 ± 0.44 μg/m^3^, O_3_ mean 98.41 ± 54.56 mg/m^3^	Age, BMI, education level, number of embryos transferred, and district fixed effect	In women under 35 years old: Biochemical pregnancy: OR = 1.004 (1.002–1.006) for O_3_, OR = 0.957 (0.938–0.976) for SO_2_ and OR = 0.992 (0.986–0.998) for NO_2_ from Gn start to oocyte retrieval; OR = 0.995 (0.992–0.999) for PM_2.5_, OR = 0.988 (0.978–0.999) for NO_2_ and OR = 0.624 (0.495–0.787) for CO from oocyte retrieval to embryo transfer; OR = 0.995 (0.992–0.998) for PM_2.5_, OR = 0.997 (0.994–0.999) for PM_10_, OR = 0.953 (0.939–0.969) for SO_2_, OR = 0.983 (0.977–0.989) for NO_2_, OR = 0.517 (0.411–0.649) for CO from embryo transfer to serum HCG test Clinical pregnancy: OR = 1.005 (1.003–1.007) for O_3_ and OR = 0.959 (0.941–0.978) for SO_2_ from Gn start to oocyte retrieval; OR = 0.996 (0.994–0.999) for PM_2.5_, OR = 1.003 (1.001–1.005) for O_3_, OR = 0.99 (0.982–0.998) for NO_2_ and OR = 0.671 (0.553–0.815) for CO from oocyte retrieval to embryo transfer; OR = 0.994 (0.99–0.998) for PM_2.5_, OR = 0.996 (0.993–0.999) for PM_10_, OR = 1.004 (1.001–1.007) for O_3_, OR = 0.946 (0.919–0.974) for SO_2_, OR = 0.979 (0.973–0.985) for NO_2_ and OR = 0.474 (0.358–0.626) for CO from embryo transfer to serum HCG test
Zhang et al. 2022 [49]	China, 2015–2019	Cohort study (12,665)	Clinical records from the Center for Reproductive Medicine, Ren Ji Hospital, School of Medicine, Shanghai Jiao Tong University	IVF and ICSI with fresh or frozen embryos (30.35 ± 4.1)	Live birth; Biochemical pregnancy; Clinical pregnancy	90 days prior to oocyte retrieval; one year prior to oocyte retrieval; the day of oocyte retrieval to the serum hCG test or the end of the pregnancy; one year prior to oocyte retrieval to the serum hCG test or the end of the pregnancy	PM_10_, PM_2.5_, SO_2_, NO_2_, CO and O_3_	The data were provided by Shanghai Meteorological Bureau Yangtze River Delta Center, which were obtained from 149 national-standard monitoring stations of China National Environmental Monitoring Center	Available data from the nearest monitoring station were applied to estimate the concentrations of air pollutants for each participant	PM_2.5_ mean (41.85 to 44.79 μg/m^3^); PM_10_ mean (64.37 to 70.02 μg/m^3^); CO mean (0.78 to 0.81 mg/m^3^); NO_2_ mean (39.79 to 40.56 μg/m^3^); SO_2_ mean (13.04 to 15.51 μg/m^3^); O_3_ mean (213.61 to 262.06 μg/m^3^)	Female age, BMI, employment status, education level, type of embryo transfer, number of embryos transferred, stimulation protocols, fertilization method, number of oocytes retrieved, and endometrial thickness	Biochemical pregnancy: OR = 0.92 (0.87–0.97) for PM_10_, OR = 0.91 (0.86–0.96) for PM_2.5_, OR = 0.93 (0.90, 0.97) for SO_2_, OR = 0.95 (0.91–0.99) for CO 90 days prior to oocyte retrieval; OR = 0.85 (0.81–0.90) for PM_10_, OR = 0.79 (0.74–0.84) for PM_2.5_, OR = 0.85 (0.82–0.89) for SO_2_, OR = 0.86 (0.84–0.93) for CO, OR = 1.14 (1.08–1.20) for O_3_ one year prior to oocyte retrieval Live birth; OR = 0.95 (0.90–1.00) for PM_10_, OR = 0.94 (0.89–0.98) for PM_2.5_, OR = 0.93 (0.90–0.97) for SO_2_ and 0.95 (0.91–0.99) for CO 90 days prior to oocyte retrieval; OR = 0.89 (0.84–0.93) for PM_10_, OR = 0.82 (0.77–0.87) for PM_2.5_, OR = 0.87 (0.83–0.91) for SO_2_, OR = 0.91 (0.87–0.96) for CO, OR = 1.13 (1.07–1.19) for O_3_ one year prior to oocyte retrieval Clinical pregnancy: OR = 0.95 (0.90, 0.99) for PM_2.5_, OR = 0.94 (0.91, 0.98) for SO_2_, OR = 0.95 (0.91, 0.99) for CO 90 days prior to oocyte retrieval; OR = 0.86 (0.82–0.90) for PM_10_, OR = 0.80 (0.75–0.85) for PM_2.5_, OR = 0.86 (0.82–0.90) for SO_2_, OR = 1.00 (0.96–1.04) for NO_2_, OR = 0.90 (0.86–0.94) for CO, OR = 1.14 (1.08–1.20) for O_3_ one year prior to oocyte retrieval

*Abbreviations*: *CO* Carbon monoxide, *NO*_*2*_ Nitrogen dioxide, *O*_*3*_ Ozone, *PM*_*2.5*_ Fine particulate matter, *PM*_*10*_ Inhalable particulate matter, *SO*_*2*_ Sulfur dioxide, *ART* Assisted reproductive technology, *ICSI* Intracytoplasmic sperm injection, *IVF* In vitro fertilization, *FET* Frozen-hawed embryo transfer, *AQI* Air Quality Index, *ppb* Parts per billion, *OR* Odd ratio, *RR* Relative ratio, *HR* Hazard ratio, *Gn* Gonadotrophin, *NOx* Nitrogen oxide, *BC* Black carbon

### Risk of bias in individual studies

In our systematic review and meta-analysis, the risk of bias for individual studies was evaluated using the NTP/OHAT Risk of Bias Rating Tool. This tool was chosen for its comprehensive coverage of critical domains relevant to our study types, including selection bias, confounding bias, detection bias for exposure characterization and outcome assessment, attrition/exclusion bias, selective reporting bias, and conflict of interest [50, 51]. Each domain was independently assessed for each included study by two authors (JCQ and MYZ), who evaluated and rated the potential risk on a four-point scale (definitely low, probably low, probably high, or definitely high). Based on these ratings, the overall study quality was then classified into one of three tiers [52]. Any disagreements in ratings between the two authors were resolved through discussion or, when necessary, consultation with a third author (CYH). The results of the risk of bias assessment played a pivotal role in interpreting our results, providing crucial context for understanding the robustness and reliability of the evidence base. (detailed questions and the rationale for the assessment of each study can be found in Appendix 3).

### Confidence in the body of evidence

In our systematic review and meta-analysis, we employed the National Toxicology Program's Office of Health Assessment and Translation (NTP/OHAT) framework [53] supported by the GRADE approach [54, 55] to assess the quality of evidence for each outcome across the included studies. Appendix 4 provides additional details on this evaluation process. The GRADE approach enables a comprehensive assessment of the confidence in the body of evidence, considering various factors that can either enhance (such as large effect size, dose–response relationship, consistency across different study designs, populations, or species, and thorough consideration of confounding factors) or diminish (such as risk of bias, inconsistency, indirectness, imprecision, and publication bias) the initial level of confidence. Due to the potential for unmeasured confounding in observational studies, our initial confidence level was considered moderate. However, by carefully evaluating these factors, we assigned an overall quality rating of “High”, “Moderate”, “Low”, or “Very Low” to each exposure-outcome combination. A rating of "High" quality indicates substantial confidence that the true effect is close to the estimated effect, while a rating of “Very Low” quality reflects minimal confidence in the effect estimate, suggesting that the true effect is likely to deviate significantly. Through this rigorous evaluation process, we aim to provide a comprehensive and reliable assessment of the evidence, ensuring that the quality of each exposure-outcome combination is appropriately conveyed.

### Data synthesis and meta-analysis

The random effects model was used to meta-analyze the risk estimates across these studies, considering clinical heterogeneity existed across them [33, 56, 57]. We chose single-pollutant models for studies that reported effect values across multiple sets of adjusted models. Based on the assumption that all hazard ratios (HR), relative risks (RR), and odds ratios (OR) were comparable, all three risk estimates were included in a meta-analysis. This is acceptable in the current situation where outcomes of interest are common while effect sizes are small [58]. Within each specific assisted reproduction procedure, we consider that the combination of specific air pollutant exposure and pregnancy outcome, including at least two effect values, was the minimum number to perform a meta-analysis. When the desired effect values were only available in the studied graph, we used Origin 2021 to extract them from the graph. Using the WHO conversion factor between parts per billion (ppb) and µg/m^3^ of air pollution, we converted the study to the same metric (1 ppb = 1.15 µg/m^3^ for CO, 1 ppb = 1.88 µg/m^3^ for NO_2_, 1 ppb = 1.96 µg/m^3^ for O_3_, 1 ppb = 2.62 µg/m^3^ for SO_2_) [59]. Where quartiles of exposure were used in the study, we calculated the difference between the mean of the first and fourth quartiles, arguing that the estimated effect was specific to this difference to convert them into data with continuous meaning [60]. Then, for continuous exposure, depending on the type of exposure (standard deviation, IQR, unit increment, or converted categorical exposure data) used in each study, we performed different conversion methods to make the increments consistent across air exposures. The meta-analysis input data were RRs of standardized increments of air pollutant concentrations (10 µg/m^3^ for PM_2.5_, PM_10_, NO_2_, O_3_, and SO_2_), except for the standardized increment of 0.5 mg/m^3^ for CO [61], using the following formulas [62]:

$${RR}_{\left(standardized\right)}= {RR}_{(original)}^{Increment(10 or 0.5)/Increment(original)}$$

In this study, heterogeneity across studies was assessed using Cochran's Q test (with a p-value less than 0.1 indicating statistical significance) and the I^2^ statistic. The I^2^ values were categorized as follows: 0–25% represented low heterogeneity, 25–50% represented moderate heterogeneity, and 50–100% represented substantial heterogeneity [63]. Funnel plots were used to visually investigate publication (small study) bias. Since the number of studies was less than ten, only the results from any period of assisted reproductive process exposure are presented [64]. Egger's test was employed to objectively assess evidence of asymmetry in the funnel plots [65]. Sensitivity analysis was conducted by omitting one study at a time and then performing a meta-analysis of the overall effect values. All analyses were performed using R software (version 4.2.1), and p-values less than 0.05 for two-sided tests were considered statistically significant.

## Results

### Study selection and characteristics

The literature search process is visually represented by the PRISMA flow chart, available in Appendix 1. Our search strategy yielded a total of 439 unique studies. Following the removal of duplicates and studies not relevant to the topic, a final selection of 44 studies was identified for comprehensive full-text evaluation. Among these, 12 studies were included in the meta-analysis [6, 18, 19, 21, 26, 34, 35, 41, 44, 46–48] and 8 studies were selected for systematic review by descriptive analysis. Zeng et al. 2020 [20] did not include the reported increase in units exposed when conducting their statistical analysis. Furthermore, three studies focused on investigating short-term ambient air pollution (e.g., time-series studies within two weeks before and after oocyte retrieval, and study where the exposure window was within 14 days of the last menstrual period) [39, 40, 42]. Additionally, three studies had subdivision periods of the ART process that did not align with the timeframe of the main study [22, 43, 49]. Lastly, one study employed machine learning statistical methods, which resulted in the unavailability of effect estimates for meta-analysis [45].

**Fig. 1:**
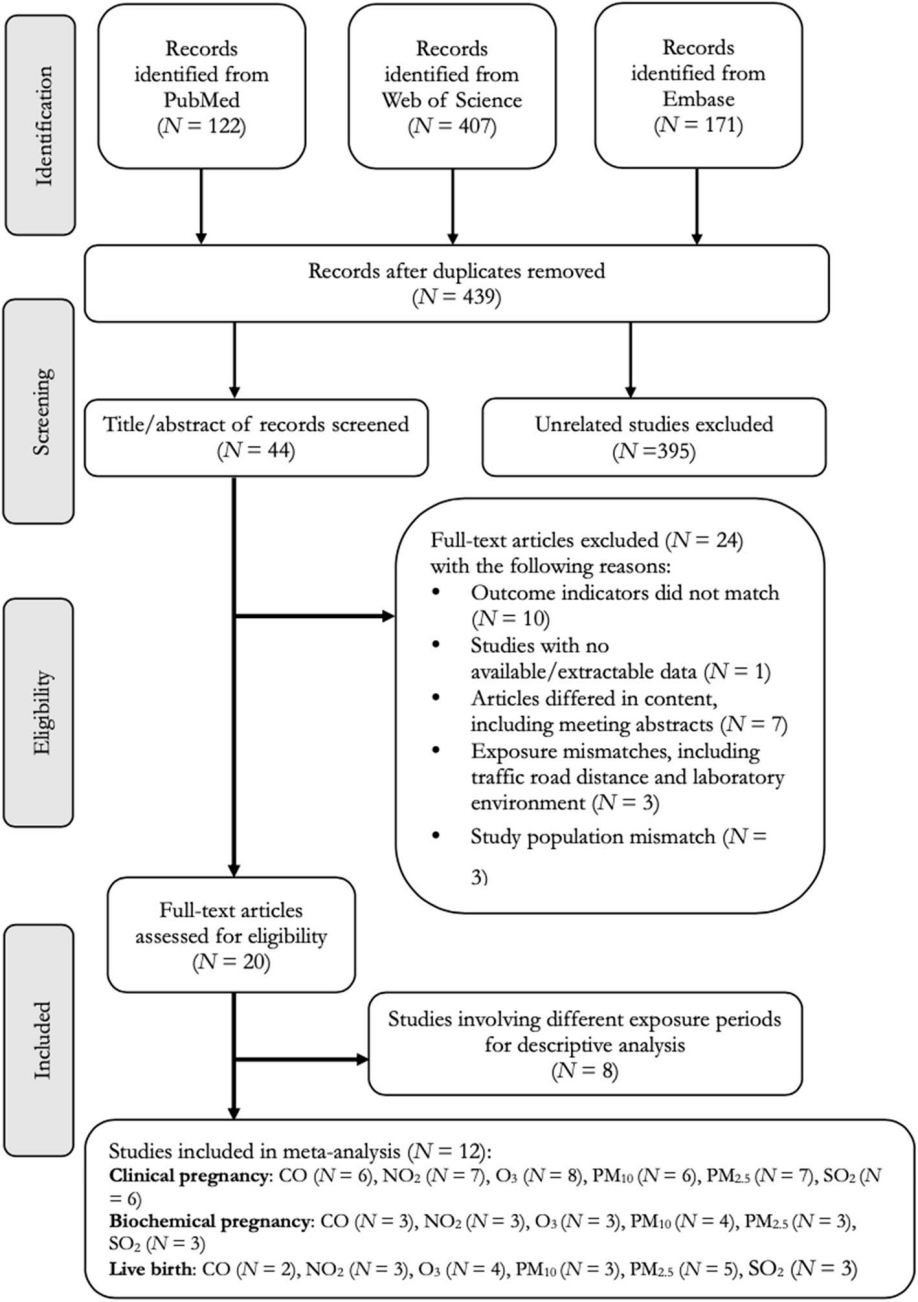
Flow chart of the study selection process

Figure 1 elucidates the criteria for the exclusion and inclusion of the studies examined. Table 1 presents the principal characteristics of these included studies. Of these, eleven were conducted in China, three in the United States, and one each in South Korea, Spain, Italy, Brazil, and France. The studies, all designed as cohorts, exhibited a varied study population, with participant ages ranging from 19 to 49 years. Additionally, the studies spanned distinct periods, with initiation dates ranging from 2000 to 2020. Among the varied outcomes studied, clinical pregnancy emerged as the most prevalent, being the focus of eight studies.

### Risk of bias assessment

Table. S2 and Appendix 5 present a comprehensive overview of the risk of bias assessment conducted for all the studies included in our meta-analyses. Out of the 20 studies examined, 8 were deemed to have a "probably low risk of confounding bias," while 11 studies were identified as having a “probably high risk of confounding bias”. The high risk classification was attributed to inadequate adjustment for socio-economic factors and the absence of BMI adjustment, which is a crucial confounding variable. Regarding detection bias, more than half of the studies (*N* = 13) were classified as having a “probably high risk of detection bias” due to the reliance on data from the nearest air monitoring station to the self-reported address [66]. However, for outcome assessment, the outcomes of interest were validated through laboratory tests, ensuring a low risk of bias. Selective reporting bias was not observed as all pre-defined outcomes were reported, resulting in a classification of “probably low risk of bias”. Moreover, there was no evidence of missing outcome data or incomplete follow-up across the studies. Selection bias was deemed “probably low risk of bias” as the studies were retrospective cohorts with both exposed and non-exposed groups selected from the same eligible population using uniform ascertainment methods and inclusion/exclusion criteria, independent of health status. All studies included in the analysis were publicly funded, and no conflicts of interest were reported by any of the authors. In summary, based on the overall assessment, all studies were categorized as either Tier 1 (*N* = 2) or Tier 2 (*N* = 18), indicating the presence of plausible bias that raises some doubts about the obtained results.

### Data synthesis and meta-analysis

#### Ambient air pollution and clinical pregnancy

**Fig. 2:**
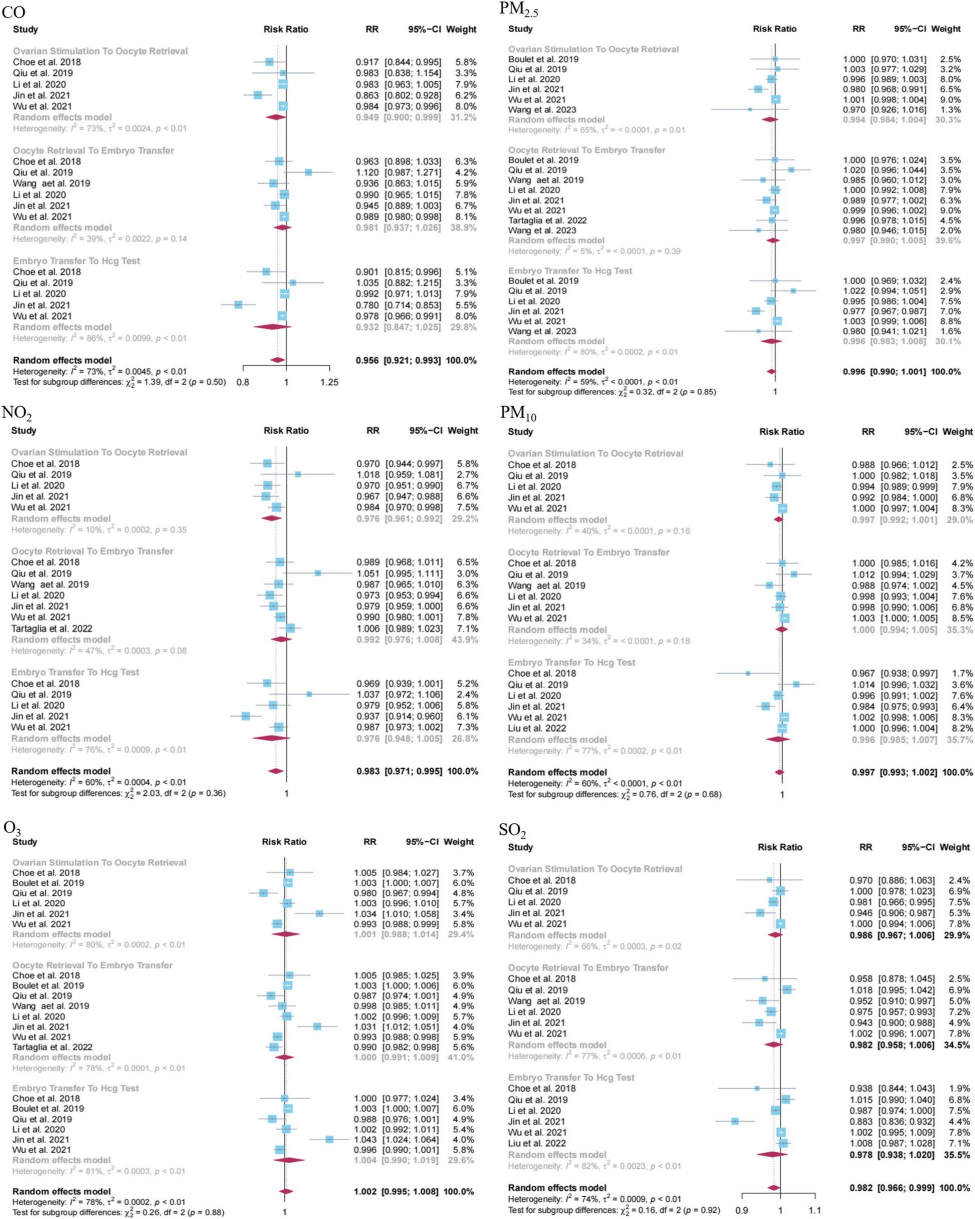
Forest plot of the association between exposure to ambient air pollution and clinical pregnancy during different stages of assisted reproductive process

Eight studies investigated the associations between exposure to various air pollutants such as CO (*N* = 6), NO_2_ (*N* = 7), O_3_ (*N* = 8), PM_10_ (*N* = 6), PM_2.5_ (*N* = 7), SO_2_ (*N* = 6) with clinical pregnancy. Our meta-analysis findings revealed that exposure to CO (RR = 0.949, 95% CI: 0.900, 0.999; I^2^ = 73%) and NO_2_ (RR = 0.976, 95% CI: 0.961, 0.992; I^2^ = 10%) during the ovarian stimulation to oocyte retrieval period were inversely associated with the incidence of clinical pregnancy. In contrast, other types of ambient air pollution and the remaining analyzed pollution-outcome pairs were found to be statistically non-significant (Fig. 2). Moreover, our data showed that exposure to CO (RR = 0.956, 95% CI: 0.921, 0.993; I^2^ = 73%), NO_2_ (RR = 0.983, 95% CI: 0.971, 0.995; I^2^ = 60%) and SO_2_ (RR = 0.982, 95% CI: 0.966, 0.999; I^2^ = 74%) at any stage of the assisted reproductive process might lead to a decrease in the incidence of clinical pregnancy.

Sensitivity analyses revealed that excluding two individual studies influenced the stability of results concerning CO exposure from ovarian stimulation to oocyte retrieval. However, the outcomes were largely consistent when the same method was applied to the rest of the air pollutant-outcome pairs (Appendix 6). Our funnel plots suggest potential publication bias in the results related to exposure to air pollutants at any phase of the assisted reproductive process (Fig. S1, Appendix 5). To further scrutinize this publication bias for each air pollutant-outcome pair during specific exposure periods, we conducted an Egger’s test. The results revealed a publication bias only in relation to SO_2_ exposure during any phase of the assisted reproductive process and clinical pregnancy outcome (*P* = 0.0095).

#### Ambient air pollution and biochemical pregnancy

**Fig. 3:**
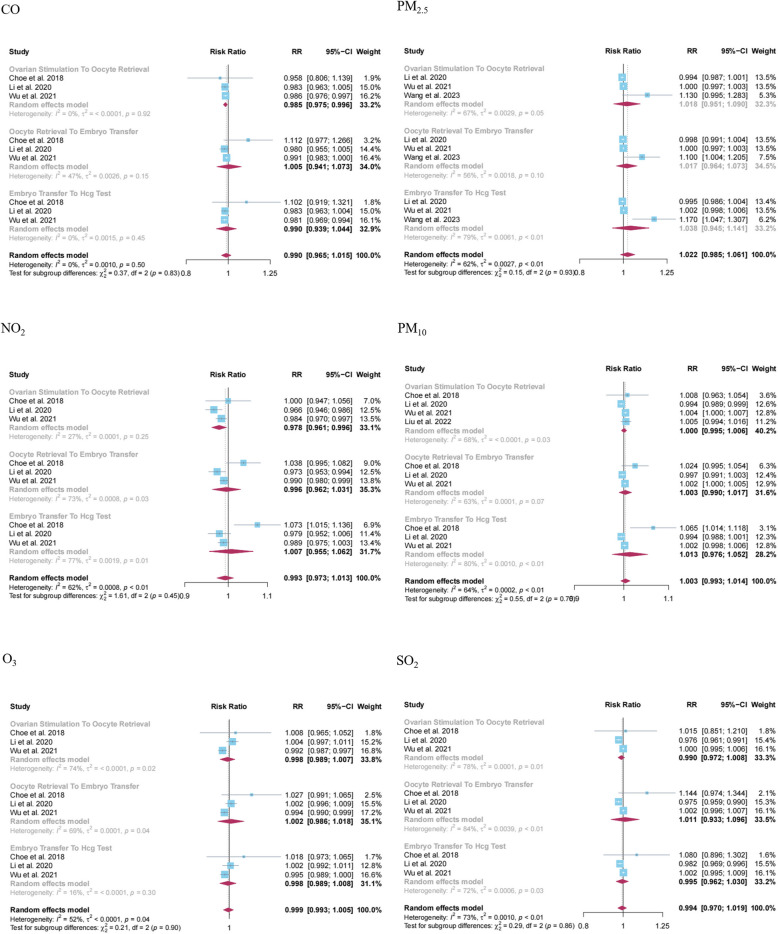
Forest plot of the association between exposure to ambient air pollution and biochemical pregnancy during different stages of assisted reproductive process

A total of four studies were analyzed, investigating the associations between exposure to several air pollutants such as CO (*N* = 3), NO_2_ (*N* = 3), O_3_ (*N* = 3), PM_10_ (*N* = 4), PM_2.5_ (*N* = 3), SO_2_ (*N* = 3) with biochemical pregnancy. Our meta-analysis findings indicated that exposure to CO (RR = 0.985, 95% CI: 0.975, 0.996; I^2^ = 0%) and NO_2_ (RR = 0.978, 95% CI: 0.961, 0. 996; I^2^ = 27%) during the ovarian stimulation to oocyte retrieval period were inversely associated with the incidence of biochemical pregnancy. Other types of ambient air pollutants and the remaining analyzed air pollutant-outcome pairs were found to be statistically non-significant (Table 2). The results of the forest plots are illustrated in Fig. 3. Sensitivity analysis indicated that the summary results of this meta-analysis were affected when the study by [48] was excluded (Appendix 6). However, based on funnel plots and Egger’s tests, no evidence of publication bias was detected in these analyses (Fig. S2 and Appendix 5).

**Table 2:** Overall meta-analytical summary estimates of associations between exposure to ambient air pollution and pregnancy outcome in women treated with assisted reproductive technologies

Air pollutant	Exposure period	No. of studies included	RR (95% CI)	I^2^ (%)	Tau^2^	*P*-value for Cochran’s Q test	*P*-value for egger’s test
**Clinical pregnancy**							
**CO**	Any period of assisted reproductive process	16	**0.956 (0.921, 0.993)**	73	0.0045	0	0.0537
	Ovarian stimulation to oocyte retrieval	5	**0.949 (0.900, 0.999)**	73	0.0024	0.005	0.1795
	Oocyte retrieval to embryo transfer	6	0.981 (0.937, 1.026)	39	0.0022	0.143	0.6776
	Embryo transfer to hCG test	5	0.932 (0.847, 1.025)	86	0.0099	0	0.3386
**NO**_**2**_	Any period of assisted reproductive process	17	**0.983 (0.971, 0.995)**	60	0.0004	0.001	0.9549
	Ovarian stimulation to oocyte retrieval	5	**0.976 (0.961, 0.992)**	10	0.0002	0.347	0.803
	Oocyte retrieval to embryo transfer	7	0.992 (0.976, 1.008)	47	0.0003	0.077	0.5619
	Embryo transfer to hCG test	5	0.976 (0.948, 1.005)	76	0.0009	0.002	0.9728
**O**_**3**_	Any period of assisted reproductive process	20	1.002 (0.995, 1.008)	78	0.0002	0	0.9692
	Ovarian stimulation to oocyte retrieval	6	1.001 (0.988, 1.014)	80	0.0002	0	0.9954
	Oocyte retrieval to embryo transfer	8	1.000 (0.991, 1.009)	78	0.0001	0	0.8468
	Embryo transfer to hCG test	6	1.004 (0.990, 1.019)	81	0.0003	0	0.8122
**PM**_**10**_	Any period of assisted reproductive process	17	0.997 (0.993, 1.002)	60	0.0001	0.001	0.0643
	Ovarian stimulation to oocyte retrieval	5	0.997 (0.992, 1.001)	40	0.0000	0.158	0.3606
	Oocyte retrieval to embryo transfer	6	1.000 (0.994, 1.005)	34	0.0000	0.185	0.3812
	Embryo transfer to hCG test	6	0.996 (0.985, 1.007)	77	0.0002	0.001	0.3272
**PM**_**2.5**_	Any period of assisted reproductive process	20	0.996 (0.990, 1.001)	59	0.0001	0	0.1167
	Ovarian stimulation to oocyte retrieval	6	0.994 (0.984, 1.004)	65	0.0000	0.014	0.2295
	Oocyte retrieval to embryo transfer	8	0.997 (0.990, 1.005)	5	0.0001	0.391	0.5427
	Embryo transfer to hCG test	6	0.996 (0.983, 1.008)	80	0.0001	0.01	0.5350
**SO**_**2**_	Any period of assisted reproductive process	17	**0.982 (0.966, 0.999)**	74	0.0009	0	0.0095
	Ovarian stimulation to oocyte retrieval	5	0.986 (0.967, 1.006)	66	0.0003	0.018	0.1794
	Oocyte retrieval to embryo transfer	6	0.982 (0.958, 1.006)	77	0.0006	0.001	0.1557
	Embryo transfer to hCG test	6	0.978 (0.938, 1.020)	82	0.0023	0	0.2698
**Biochemical pregnancy**							
**CO**	Any period of assisted reproductive process	9	0.990 (0.965, 1.015)	0	0.0010	0.499	0.4997
	Ovarian stimulation to oocyte retrieval	3	**0.985 (0.975, 0.996)**	0	0.0000	0.922	0.1275
	Oocyte retrieval to embryo transfer	3	1.005 (0.941, 1.073)	47	0.0026	0.152	0.6783
	Embryo transfer to hCG test	3	0.990 (0.939, 1.044)	0	0.0015	0.453	0.148
**NO**_**2**_	Any period of assisted reproductive process	9	0.993 (0.973, 1.013)	62	0.0008	0.006	0.3015
	Ovarian stimulation to oocyte retrieval	3	**0.978 (0.961, 0.996)**	27	0.0001	0.254	0.9368
	Oocyte retrieval to embryo transfer	3	0.996 (0.962, 1.031)	73	0.0008	0.025	0.7242
	Embryo transfer to hCG test	3	1.007 (0.955, 1.062)	77	0.0019	0.013	0.5097
**O**_**3**_	Any period of assisted reproductive process	9	0.999 (0.993, 1.005)	52	0.0001	0.035	0.0575
	Ovarian stimulation to oocyte retrieval	3	0.998 (0.989, 1.007)	74	0.0000	0.022	0.7135
	Oocyte retrieval to embryo transfer	3	1.002 (0.986, 1.018)	69	0.0001	0.04	0.3658
	Embryo transfer to hCG test	3	0.998 (0.989, 1.008)	16	0.0000	0.303	0.3204
**PM**_**10**_	Any period of assisted reproductive process	10	1.003 (0.993, 1.014)	64	0.0002	0.003	0.5741
	Ovarian stimulation to oocyte retrieval	4	1.000 (0.995, 1.006)	68	0.0000	0.026	0.9922
	Oocyte retrieval to embryo transfer	3	1.003 (0.990, 1.017)	63	0.0001	0.065	0.8619
	Embryo transfer to hCG test	3	1.013 (0.976, 1.052)	80	0.0010	0.008	0.6579
**PM**_**2.5**_	Any period of assisted reproductive process	9	1.022 (0.985, 1.061)	62	0.0027	0.01	0.1386
	Ovarian stimulation to oocyte retrieval	3	1.018 (0.951, 1.090)	67	0.0029	0.05	0.7804
	Oocyte retrieval to embryo transfer	3	1.017 (0.964, 1.073)	56	0.0018	0.10	0.4880
	Embryo transfer to hCG test	3	1.038 (0.945, 1.141)	79	0.0061	0.01	0.6289
**SO**_**2**_	Any period of assisted reproductive process	9	0.994 (0.970, 1.019)	73	0.0010	0	0.5796
	Ovarian stimulation to oocyte retrieval	3	0.990 (0.972, 1.008)	78	0.0001	0.01	0.6974
	Oocyte retrieval to embryo transfer	3	1.011 (0.933, 1.096)	84	0.0039	0.002	0.9537
	Embryo transfer to hCG test	3	0.995 (0.962, 1.030)	72	0.0006	0.027	0.9106
**Live birth**							
**CO**	Any period of assisted reproductive process	4	**0.975 (0.963, 0.988)**	36	0.0001	0.195	0.4044
	Ovarian stimulation to oocyte retrieval	2	0.981 (0.955, 1.008)	0	0.0002	0.380	NA
	Oocyte retrieval to embryo transfer	1	**0.975 (0.961, 0.989)**	NA	NA	NA	NA
	Embryo transfer to hCG test	1	**0.966 (0.950, 0.981)**	NA	NA	NA	NA
**NO**_**2**_	Any period of assisted reproductive process	7	**0.954 (0.916, 0.995)**	80	0.0027	0	0.0016
	Ovarian stimulation to oocyte retrieval	2	0.940 (0.854, 1.035)	88	0.2243	0.001	NA
	Oocyte retrieval to embryo transfer	3	0.977 (0.939, 1.017)	68	0.0010	0.045	0.3445
	Embryo transfer to hCG test	2	0.928 (0.818, 1.053)	92	0.0077	0.001	NA
**O**_**3**_	Any period of assisted reproductive process	10	1.008 (0.995, 1.021)	77	0.0004	0	0.5813
	Ovarian stimulation to oocyte retrieval	3	1.015 (0.978, 1.054)	89	0.0010	0	0.7811
	Oocyte retrieval to embryo transfer	4	1.000 (0.993, 1.007)	54	0.0000	0.09	0.6478
	Embryo transfer to hCG test	3	1.015 (0.984, 1.047)	84	0.0007	0.002	0.6927
**PM**_**10**_	Any period of assisted reproductive process	9	1.002 (0.989, 1.014)	33	0.0003	0.151	0.1169
	Ovarian stimulation to oocyte retrieval	3	1.011 (0.973, 1.050)	68	0.0009	0.044	0.0654
	Oocyte retrieval to embryo transfer	3	0.998 (0.990, 1.006)	21	0.0000	0.282	0.5657
	Embryo transfer to hCG test	3	1.003 (0.985, 1.020)	0	0.0002	0.443	0.451
**PM**_**2.5**_	Any period of assisted reproductive process	14	0.997 (0.989, 1.005)	0	0.0001	0.780	0.3199
	Ovarian stimulation to oocyte retrieval	5	0.999 (0.990, 1.009)	0	0.0000	0.880	0.8255
	Oocyte retrieval to embryo transfer	5	0.994 (0.980, 1.008)	0	0.0001	0.563	0.0575
	Embryo transfer to hCG test	4	0.998 (0.981, 1.014)	0	0.0001	0.500	0.1875
**SO**_**2**_	Any period of assisted reproductive process	7	0.998 (0.989, 1.006)	28	0.0001	0.216	0.0193
	Ovarian stimulation to oocyte retrieval	2	1.001 (0.994, 1.007)	0	0.0001	0.910	NA
	Oocyte retrieval to embryo transfer	3	0.992 (0.971, 1.014)	68	0.0003	0.044	0.2962
	Embryo transfer to hCG test	2	0.998 (0.988, 1.008)	48	0.0001	0.170	NA

Summary effect estimates are in bold when the 95% CI do not include 1. For the meta-analysis on any period of assisted reproductive process, we did not extract the data corresponding to the specific period from ovarian stimulation to hCG test to avoid the repeated effect estimates entrance

*Abbreviations*: *CO* Carbon monoxide, *NO*_*2*_ Nitrogen dioxide, *O*_*3*_ Ozone, *PM*_*2.5*_ Fine particulate matter, *PM*_*10*_ Inhalable particulate matter, *SO*_*2*_ Sulfur dioxide, *NA* Not applicable, *hCG* Human chorionic gonadotropin, *RR* Relative risk

#### Ambient air pollution and live birth

**Fig. 4:**
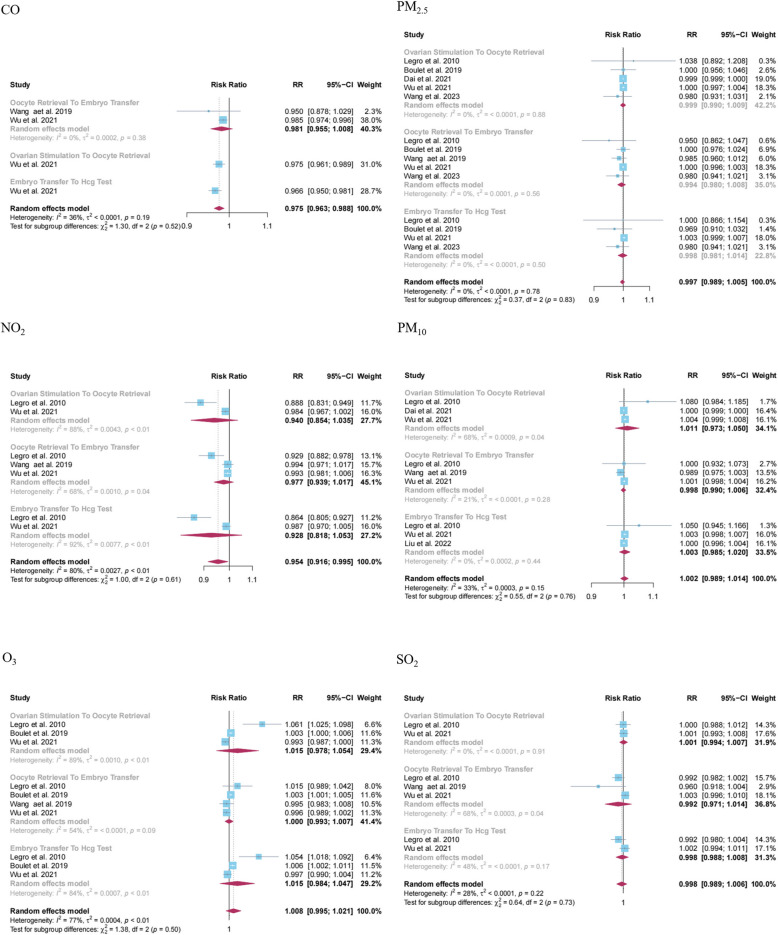
Forest plot of the association between exposure to ambient air pollution and live birth during different stages of assisted reproductive process

The meta-analysis included 5 studies to examine the associations between exposure to various pollutants, including CO (*N* = 2), NO_2_ (*N* = 3), O_3_ (*N* = 4), PM_10_ (*N* = 3), PM_2.5_ (*N* = 5), SO_2_ (*N* = 3) with live birth. The results indicated that exposure to CO (RR = 0.956, 95% CI: 0.921, 0.993; I^2^ = 73%) and NO_2_ (RR = 0.983, 95% CI: 0.971, 0.995; I^2^ = 60%) during any phase of the assisted reproduction process seemed to reduce the incidence of live birth (Table 2). The corresponding forest plot outcomes are presented in Fig. 4. Despite these findings, the conclusions require further validation due to sensitivity analyses demonstrating variability in NO_2_ exposure results, and both funnel plots and Egger’s tests revealing a notable publication bias (Fig. S3 and Appendix 5).

### Confidence in the body of evidence and level of evidence

Table. S3 and Appendix 5 presents the summary of confidence ratings for the included studies (*N* = 72). According to the NTP/OHAT framework, only experimental and controlled studies are initially assigned a “high confidence” rating. This rating ensures the elimination of random allocation bias and confirms that exposure precedes the onset of the outcome. Since all the studies included in this review were retrospective cohort studies, an initial rating of “moderate confidence” was allocated, considering the inherent limitations of such study designs.

Regarding downgrading factors, we evaluated a number of elements to potentially decrease the initial confidence rating, which included the risk of bias or unexplained inconsistency. The NTP/OHAT guidelines suggest reserving downgrading due to risk of bias for instances where the risk is significant across the majority of studies comprising the evidence body. As most evidence was categorized under Tier 2 and Tier 1, with no studies under Tier 3, we judged the risk of bias to be insufficient to undermine confidence in the results. The primary downgrading factor for some exposure-outcome pairs was inconsistency, owing to substantial heterogeneity across studies (I^2^ > 50%). However, confidence intervals did not negatively impact the confidence rating as all upper-to-lower 95%CI ratios were far from the proposed threshold of 10 for penalization consideration, thereby regarded as “unlikely imprecision”. Publication bias was noticeable for three exposure-outcome pairs as there was a statistically significant test for small study effects (Egger’s test *P*-value < 0.05). Directness was not compromised as all studies were human studies and the follow-up time was sufficient to develop the outcome of interest. Concerning upgrading factors, several elements were also considered to potentially enhance the confidence rating. Residual bias was deemed the most significant of these factors due to previously noted methodological differences across studies that might constrain absolute risk estimation. However, this factor was considered insufficient for upgrading support. The absence of clear monotonic or non-monotonic responses precluded the consideration of dose–response relationships, either on an individual study basis or across studies, as a factor supporting an increase in confidence. Furthermore, the available data did not allow for a comprehensive dose–response meta-analysis to be conducted. Additionally, there was no large magnitude of association as the meta-analytical effect estimates were less than 2.

In conclusion, the confidence in the body of evidence for some exposure-outcome pairs was downgraded to “low” due to inconsistency across studies and publication bias. As such, the level of evidence for a positive association between air pollution exposure and outcomes of interest was categorized as “low” or “moderate” (Table. S3 and Appendix 5). For the remaining exposure-outcome pairs that did not demonstrate statistically significant associations, the level of evidence for health effects was deemed “inadequate” following the NTP/OHAT guidelines.

## Discussion

We performed this updated systematic review and meta-analysis to assess the effects of ambient air pollution exposure and on pregnancy outcomes in women treated with ART. Overall, this study provides evidence that exposure to CO and NO_2_ during ART procedures can decrease the rates of both clinical and biochemical pregnancy, particularly during the phase from ovarian stimulation to oocyte retrieval. Our study was overall consistent with the recent systematic review and meta-analysis [33], which reported that air pollution exposure is associated with reduced odds of clinical pregnancy, biochemical pregnancy, and live birth. Being an updated study, we included 20 studies compared to their 14, offering a broader perspective. In addition, we conducted a thorough risk of bias assessment and provided detailed evaluations of evidence levels for each exposure-outcome combination, strengthening the credibility and comprehensiveness of our analysis. In addition, Seli et al. reviewed the available evidence and found that air pollution was associated with reduced pregnancy rates in infertile patients undergoing IVF treatments [67]. Conforti et al. also noted that air pollution reduces conception rate after spontaneous intercourse and live birth rate after IVF procedures after reviewing the literature [68]. These two reviews provide results similar to our conclusions, although without a meta-analysis of the evidence.

Inconsistencies may arise across studies investigating the same ambient air pollution due to factors such as disparities in sample size, exposure assessment, analytical methods, study populations, and variations in ART protocols across different reproductive centers. These elements potentially account for the observed heterogeneity in the examined exposure-outcome combinations. Variations were detected in the age ranges of the populations across the included studies. Despite individual studies adjusting for age as a confounding factor, residual disparities between studies may still exist, potentially contributing to the heterogeneity. Subsequent studies may seek to delve deeper into the potential relationship between pregnancy outcomes and ambient air pollution within distinct age brackets of patients undergoing ART.

In the present study, to mitigate the possible bias introduced by fresh and frozen embryos on the outcomes [69, 70], we opted to perform the meta-analysis solely on studies involving participants who received fresh cycle treatments, given that data on frozen embryos was not sufficiently robust for further analysis. Furthermore, while most studies accounted for the number of embryos transferred, a few did not. Participants may have undergone a single cycle or have had multiple embryos transferred. Given the number of studies included in our review, we did not categorize data based on the number of embryos transferred, which might introduce another potential source of heterogeneity in these analyzed exposure-outcome pairs, given the evidence of variable impacts of the number of transferred embryos on pregnancy outcomes [71, 72].

Currently, the specific biological mechanisms underpinning the association between ambient air pollution exposure and pregnancy outcomes in individuals undergoing ART are not entirely understood. It is, however, theorized that ambient air pollution may negatively influence the reproductive system through oxidative stress induction [73], inflammatory responses, endocrine disruption [74], and epigenetic changes [75]. Particulate matter and nitrogen oxides, prominent components of air pollution, can incite oxidative stress within the body, leading to cellular and tissue damage [68, 76]. This oxidative stress could compromise the quality and function of reproductive cells, such as oocytes and sperm, and potentially hinder successful embryo implantation and development [73, 77]. Moreover, air pollutants can provoke an inflammatory response, which may negatively impact reproductive health [78, 79]. Notably, inflammation has been associated with diminished ovarian function, inferior oocyte quality, and impaired embryo development [80, 81]. Air pollution exposure can also engender epigenetic modifications, potentially altering gene expression patterns in reproductive cells and embryos, consequently impairing their quality, function, and potentially leading to a decrease in ART success rates [82, 83]. Furthermore, air pollution may disturb the body's endocrine equilibrium, disrupting hormonal balance, and thereby negatively impacting women's reproductive health [74, 84]. To fully comprehend the relevant mechanisms in humans, further research is crucial.

This study suggests that the different stages of ART are variably affected by ambient air pollution exposure, the impact of which may differ depending on the specific ART stage and type of ambient air pollutant. The period from ovarian stimulation to oocyte retrieval is particularly susceptible to ambient air pollution, potentially due to oxidative stress and inflammation of the ovaries caused by pollutants. This could disrupt follicular growth and maturation, thus reducing the quality and quantity of oocytes retrieved [85–88]. Furthermore, air pollution may induce inflammation and oxidative stress in the reproductive tract, damaging both sperm and oocytes, and undermining their binding capacity, thereby impairing fertilization [89, 90]. As the embryo attaches to the endometrium and begins growth, inflammation and epigenetic alterations may decrease the likelihood of successful implantation and impact proper embryonic development [91–93]. Specifically, CO has been demonstrated to reduce estrogen and progesterone production, disrupt the menstrual cycle, and decrease ovarian function [94, 95]. NO_2_ incites oxidative stress and inflammation, causing cellular and tissue damage, and may be associated with decreased ovarian function, poor oocyte quality, and impaired embryo development [96–98]. All these factors can contribute to adverse pregnancy outcomes with ART. While all air pollutants have the potential to impact female reproductive health, their specific mechanistic impacts may differ. This suggests that the specific timing of air pollution exposure may have differential effects on pregnancy outcomes in women undergoing ART treatment. Moreover, as for the biological mechanism directly related to ART, epidemiological research has revealed associations between specific air pollutants and ART outcomes such as “no pregnancy”, “miscarriage” and “clinical pregnancy”. Nevertheless, how each air pollutant content affect human oocyte and embryo quality has not been well studied according to current literature [34]. Therefore, further research is needed to explore the potential mechanisms impacting pregnancy outcomes in women undergoing ART at different periods of ambient air pollution exposure. By elucidating these mechanisms, healthcare providers and policymakers can develop targeted interventions to mitigate the potential negative impact of air pollution on reproductive health.

This study has several strengths include meticulous categorization of ART stages to minimize inconsistencies in exposure periods. We employed robust statistical methods to transform data from highest versus lowest exposures into a continuous format and standardized units, increasing the number of studies for meta-analysis. This approach enables more realistic assessments of air pollution effects. Additionally, the enhanced risk of bias rating tool and WHO's GRADE assessment for air pollution studies were used for evaluation. The findings indicate no significant bias risk, with exposure-outcome pairs showing low to moderate quality, lending a reliable evidence base to this meta-analysis.

This study possesses several noteworthy limitations. Firstly, it is important to acknowledge that the inclusion of a relatively small number of primary studies for each combination could potentially mask the presence of publication bias. Secondly, our meta-analysis stringently grouped exposure periods but overlooked studies investigating air pollution exposure before or after ART cycle initiation, from embryo transfer to live birth phase, and short-term exposure. These studies, unable to strictly categorize exposure periods and hence excluded from the meta-analysis, could potentially reveal the true impact of ambient air pollution on ART-related pregnancy outcomes. Thirdly, potential interactions between ambient air pollutants were not accounted for, as none of our extracted data adjusted for other air pollutants. Future studies should explore how the six main air pollutants interact with each other and with meteorological factors. It is also recommended to use advanced methods in environmental epidemiology for better understanding of exposure mixtures. Techniques like toxicant scores, weighted quantile sum (WQS) regression, and Bayesian kernel machine regression (BKMR) could provide deeper insights into the combined effects of these pollutants and improve the assessment of environmental health risks. Fourthly, the limitation of included studies was the using an ecological approach for measuring air pollutants without considering individual activity patterns, occupational exposures, interactions among pollutants and other risk factors such as air temperature level. Methods for measuring air pollutants can play an important role, and future research should explore and adopt more integrated approaches. Lastly, although some differences were found in this study, the number of studies is small, the heterogeneity is large, and further research is needed. Furthermore, due to the limited number of studies available for each specific exposure and outcome combination, we did not investigate the sources of heterogeneity in our analysis. It is important to highlight the scarcity of studies conducted in this particular field, which limits the availability of data for analyzing the exposure to ambient air pollution across various age groups and factors associated with the number of embryo transfers. Therefore, a more comprehensive investigation incorporating a wider range of studies is warranted to address these aspects effectively. Furthermore, the investigation of the potential dose–response relationship in this context remains an area that requires further exploration, highlighting the imperative for future studies in this field. By examining the quantitative association between exposure to ambient air pollution and its corresponding effects on pregnancy outcomes, these studies would contribute to enhancing our understanding of the subject matter in a more comprehensive manner.

The accuracy of the assessment of exposure to environmental air pollution also affects the reliability of the results. Only a few studies utilized spatiotemporal models [41] or land use models [39] to assess individual exposure levels. However, most of the included articles (13/20) assumed that subjects' locations during the exposure period were fixed, based on the distance from their residence or clinic to government-established air quality monitoring stations, or by using average values from various monitoring stations in a specific area as indicators of exposure levels. We acknowledge this limitation as it may not fully capture individuals' daily activity patterns and actual exposure levels, potentially leading to some degree of misclassification. Future research should consider using more refined methods for assessing exposures, such as tracking based on Global Positioning System (GPS) or time-activity diaries, in order to accurately capture real-time individual exposure levels [99, 100].

In summary, we discussed the potential impact of air pollution exposure on various outcomes in ART. These outcomes include implantation rate, clinical intrauterine pregnancy, and live birth (at least one baby born alive after 20 weeks). Factors such as physical characteristics, psychosocial factors, and primary diseases of the reproductive system may also influence ART outcomes in unknown ways [101]. Additionally, women undergoing ART treatment often face their last chance for fertility, making the treatment outcome crucial for them. Successful pregnancy and live birth not only fulfill their basic desire to become mothers but also have profound psychological and social implications [102]. Furthermore, the high cost and potential health risks associated with ART make each treatment attempt stressful and filled with expectations [103]. Understanding and improving these treatment outcomes are essential for alleviating patient burden, optimizing healthcare resource allocation, enhancing treatment efficiency, and formulating evidence-based policies. By elucidating this adverse effect of ambient air pollution on reproductive health, healthcare providers and policymakers can develop targeted interventions to mitigate its potential negative impact.

## Conclusions

This study presents the comprehensive updated systematic review and meta-analysis, assessing the epidemiological evidence of ambient air pollution exposure on pregnancy outcomes in women undergoing ART, considering risk of bias and level of evidence for each exposure-outcome combination. Our pooled findings are consistent with previous studies suggesting that exposure to CO and NO_2_ during the ovarian stimulation to oocyte retrieval phase could decrease the rates of clinical and biochemical pregnancy. However, the impact of air pollution exposure on biochemical pregnancy and live birth remains less certain. Considering the potential presence of unaccounted heterogeneity and the limited number of studies included, it is essential to cautiously interpret the evidence observed in this study. Further research is necessary to address these limitations and provide a more definitive understanding.

## Supplementary Information

Supplementary Material 1: Appendix 1. Preferred Reporting Items for Systematic reviews and Meta-Analysis (PRISMA) 2009 Checklist; Appendix 2. Details for the search strategy used within each database; Appendix 3. OHAT Risk of Bias Rating Tool for Human and Animal Studies; Appendix 4. Approach to assessing the certainty of evidence from systematic reviews; Appendix 5. (Table. S2. The details the ART methodology; Table. S2. Risk of bias assessment using the National Toxicology Program's Office of Health Assessment and Translation (NTP/OHAT) tiered risk of bias approach; Table. S3. Confidence rating: assessment of body evidence; Fig. S1. Funnel plot of publication bias in reported associations between exposure to ambient air pollution and clinical pregnancy; Fig. S2. Funnel plot of publication bias in reported associations between exposure to ambient air pollution and biochemical pregnancy; Fig. S3. Funnel plot of publication bias in reported associations between exposure to ambient air pollution and live birth). Appendix 6. Sensitivity analyses of the association between ambient air pollution exposure and pregnancy outcomes in women treated with assisted reproductive technologies.

## Data Availability

The datasets used and/or analyzed during the current study are available from the corresponding author on reasonable request.
