# Supplementary material for: Association between ambient air pollution exposure and pregnancy outcomes in women treated with assisted reproductive technology: an updated systematic review and meta-analysis
Source: BMC Public Health. 2025 May 2;25:1639. doi: 10.1186/s12889-024-19301-3 (PMC12046897; doi:10.1186/s12889-024-19301-3)
Supplement: Supplementary file 1 — Supplementary Material 1: Appendix 1. Preferred Reporting Items for Systematic reviews and Meta-Analysis (PRISMA) 2009 Checklist; Appendix 2. Details for the search strategy used within each database; Appendix 3. OHAT Risk of Bias Rating Tool for Human and Animal Studies; Appendix 4. Approach to assessing the certainty of evidence from systematic reviews; Appendix 5. (Table. S2. The details the ART methodology; Table. S2. Risk of bias assessment using the National Toxicology Program's Office of Health Assessment and Translation (NTP/OHAT) tiered risk of bias approach; Table. S3. Confidence rating: assessment of body evidence; Fig. S1. Funnel plot of publication bias in reported associations between exposure to ambient air pollution and clinical pregnancy; Fig. S2. Funnel plot of publication bias in reported associations between exposure to ambient air pollution and biochemical pregnancy; Fig. S3. Funnel plot of publication bias in reported associations between exposure to ambient air pollution and live birth). Appendix 6. Sensitivity analyses of the association between ambient air pollution exposure and pregnancy outcomes in women treated with assisted reproductive technologies. [file 12889_2024_19301_MOESM1_ESM.zip › Appendix 2.docx]

### Appendix 2. Details for the search strategy used within each database

**PubMed**

((((((((((((((((((((((((((((((((((((((((((((("Air Pollution"[Mesh]) OR ("Air Pollutants"[Mesh])) OR ("Nitrogen Dioxide"[Mesh])) OR ("Sulfur Dioxide"[Mesh])) OR ("Carbon Monoxide"[Mesh])) OR ("Ozone"[Mesh])) OR ("Particulate Matter"[Mesh])) OR ("Nitrogen Oxides"[Mesh])) OR (Air Pollution[Title/Abstract])) OR (Air Pollutions[Title/Abstract])) OR (Pollution, Air[Title/Abstract])) OR (Air Quality[Title/Abstract])) OR (Air Pollutants[Title/Abstract])) OR (Air Pollutant[Title/Abstract])) OR (Air Environmental Pollutants[Title/Abstract])) OR (Environmental Air Pollutants[Title/Abstract])) OR (Nitrogen Dioxide[Title/Abstract])) OR (Dioxide, Nitrogen[Title/Abstract])) OR (Nitrogen Peroxide[Title/Abstract])) OR (Peroxide, Nitrogen[Title/Abstract])) OR (Sulfur Dioxide[Title/Abstract])) OR (Sulfurous Anhydride[Title/Abstract])) OR (Carbon Monoxide[Title/Abstract])) OR (Monoxide, Carbon[Title/Abstract])) OR (Ozone[Title/Abstract])) OR (Tropospheric Ozone[Title/Abstract])) OR (Low Level Ozone[Title/Abstract])) OR (Ground Level Ozone[Title/Abstract])) OR (Particulate Matter[Title/Abstract])) OR (Ultrafine Fibers[Title/Abstract])) OR (Ultrafine Fiber[Title/Abstract])) OR (Airborne Particulate Matter[Title/Abstract])) OR (Particulate Air Pollutants[Title/Abstract])) OR (Nitrogen Oxides[Title/Abstract])) OR (Oxides, Nitrogen[Title/Abstract])) OR (Nitrogen Oxide[Title/Abstract])) OR (Oxide, Nitrogen[Title/Abstract])) OR (PM2.5[Title/Abstract])) OR (PM10[Title/Abstract])) OR (NO2[Title/Abstract])) OR (NOx[Title/Abstract])) OR (O3[Title/Abstract])) OR (SO2[Title/Abstract])) OR (black carbon[Title/Abstract])) AND ((((((((((((((((((((((((((((((((((((((((((((((((("Reproductive techniques, assisted"[MeSH]) OR "Fertilization in vitro"[MeSH]) OR Insemination, artificial[MeSH]) OR Technique, Assisted Reproductive[Title/Abstract]) OR Techniques, Assisted Reproductive[Title/Abstract]) OR Assisted Reproductive Technics[Title/Abstract]) OR Assisted Reproductive Technic[Title/Abstract]) OR Reproductive Technic, Assisted[Title/Abstract]) OR Reproductive Technics, Assisted[Title/Abstract]) OR Technic, Assisted Reproductive[Title/Abstract]) OR Technics, Assisted Reproductive[Title/Abstract]) OR Assisted Reproductive Techniques[Title/Abstract]) OR Reproductive Technology, Assisted[Title/Abstract]) OR Assisted Reproductive Technologies[Title/Abstract]) OR Assisted Reproductive Technology[Title/Abstract]) OR Reproductive Technologies, Assisted[Title/Abstract]) OR Technologies, Assisted Reproductive[Title/Abstract]) OR Technology, Assisted Reproductive[Title/Abstract]) OR Assisted Reproductive Technique[Title/Abstract]) OR Reproductive Technique, Assisted[Title/Abstract]) OR In Vitro Fertilization[Title/Abstract]) OR In Vitro Fertilizations[Title/Abstract]) OR Test-Tube Fertilization[Title/Abstract]) OR Fertilization, Test-Tube[Title/Abstract]) OR Fertilizations, Test-Tube[Title/Abstract]) OR Test Tube Fertilization[Title/Abstract]) OR Test-Tube Fertilizations[Title/Abstract]) OR Fertilizations in Vitro[Title/Abstract]) OR Test-Tube Babies[Title/Abstract]) OR Babies, Test-Tube[Title/Abstract]) OR Baby, Test-Tube[Title/Abstract]) OR Test Tube Babies[Title/Abstract]) OR Test-Tube Baby[Title/Abstract]) OR IVF[Title/Abstract]) OR Injection, Intracytoplasmic Sperm[Title/Abstract]) OR Injections, Intracytoplasmic Sperm[Title/Abstract]) OR Intracytoplasmic Sperm Injection[Title/Abstract]) OR Sperm Injection, Intracytoplasmic[Title/Abstract]) OR Intracytoplasmic Sperm Injections[Title/Abstract]) OR ICSI[Title/Abstract]) OR Injections, Sperm, Intracytoplasmic[Title/Abstract]) OR Sperm Injections, Intracytoplasmic[Title/Abstract]) OR Eutelegenesis[Title/Abstract]) OR Eutelegeneses[Title/Abstract]) OR Artificial Insemination[Title/Abstract]) OR Artificial Inseminations[Title/Abstract]) OR Inseminations, Artificial[Title/Abstract]) OR intra uterine insemination[Title/Abstract]) OR IUI[Title/Abstract])) AND ((((((((((((((((((((((((((((((((((Live Birth[MeSH]) OR Live Births[Title/Abstract]) OR Live Birth[Title/Abstract]) OR liveborn child[Title/Abstract]) OR liveborn progeny[Title/Abstract]) OR live birth rate[Title/Abstract]) OR Pregnancy Rate[MeSH Terms]) OR Rates, Pregnancy[Title/Abstract]) OR Pregnancy Rates[Title/Abstract]) OR Rate, Pregnancy[Title/Abstract]) OR Pregnancy Rate, Live-Birth[Title/Abstract]) OR Live-Birth Pregnancy Rates[Title/Abstract]) OR Pregnancy Rate, Live Birth[Title/Abstract]) OR Pregnancy Rates, Live-Birth[Title/Abstract]) OR Rate, Live-Birth Pregnancy[Title/Abstract]) OR Rates, Live-Birth Pregnancy[Title/Abstract]) OR Live-Birth Pregnancy Rate[Title/Abstract]) OR Live Birth Pregnancy Rate[Title/Abstract]) OR clinical Pregnancy[Title/Abstract]) OR oocyte quality[Title/Abstract]) OR quality of oocyte[Title/Abstract]) OR egg quality[Title/Abstract]) OR Ovocytes quality[Title/Abstract]) OR Ovocyte quality[Title/Abstract])) OR Ovulation Induction[MeSH Terms]) OR Ovarian Stimulation[Title/Abstract]) OR Ovarian Stimulations[Title/Abstract]) OR Stimulation, Ovarian[Title/Abstract]) OR Stimulations, Ovarian[Title/Abstract]) OR poor responders[Title/Abstract]) OR ovarian response[Title/Abstract]) OR (Biochemical pregnancy[Title/Abstract])) OR (Uterine pregnancy[Title/Abstract]))

Filter Language = English

Filter Dates = [none](tel:20002019)

*Results = 122*

**Web of Science (All Databases):**

TS=((Air Pollution OR Air Pollutants OR Nitrogen Dioxide OR Sulfur Dioxide OR Carbon Monoxide OR Ozone OR Particulate Matter OR Nitrogen Oxides OR Air Pollutions OR Air Quality OR Air Pollutants OR Air Pollutant OR Air Environmental Pollutants OR Environmental Air Pollutants OR Nitrogen Dioxide OR Nitrogen Peroxide OR Sulfur Dioxide OR Sulfurous Anhydride OR Carbon Monoxide OR Ozone OR Tropospheric Ozone OR Low Level Ozone OR Ground Level Ozone OR Particulate Matter OR Ultrafine Fibers OR Ultrafine Fiber OR Airborne Particulate Matter OR Particulate Air Pollutants OR Nitrogen Oxides OR Nitrogen Oxide OR PM2.5 OR PM10 OR NO2 OR NOx OR O3 OR SO2 OR black carbon) AND (Fertilization in vitro OR Assisted Reproductive Technics OR Assisted Reproductive Technic OR Assisted Reproductive Techniques OR Assisted Reproductive Technologies OR Assisted Reproductive Technology OR Assisted Reproductive Technique OR In Vitro Fertilization OR In Vitro Fertilizations OR Test-Tube Fertilization OR Test Tube Fertilization OR Test-Tube Fertilizations OR Fertilizations in Vitro OR Test-Tube Babies OR Test Tube Babies OR Test-Tube Baby OR IVF OR Intracytoplasmic Sperm Injection OR Intracytoplasmic Sperm Injections OR ICSI OR Eutelegenesis OR Eutelegeneses OR Artificial Insemination OR Artificial Inseminations OR intra uterine insemination OR IUI) AND (Live Birth OR Live Births OR liveborn child OR liveborn progeny OR live birth rate OR Pregnancy Rate OR Pregnancy Rates OR Live-Birth Pregnancy Rates OR Live-Birth Pregnancy Rate OR Live Birth Pregnancy Rate OR clinical Pregnancy OR oocyte quality OR quality of oocyte OR egg quality OR Ovocytes quality OR Ovocyte quality OR Ovulation Induction OR Ovarian Stimulation OR Ovarian Stimulations OR poor responders OR ovarian response OR Biochemical pregnancy OR Uterine pregnancy))

Filter Language = English

Filter Dates = none

Results = *407*

**Embase**

('air pollution'/exp OR 'air pollutant'/exp OR 'nitrogen dioxide'/exp OR 'sulfur dioxide'/exp OR 'carbon monoxide'/exp OR 'ozone'/exp OR 'particulate matter'/exp OR 'nitrogen oxide'/exp OR 'Air Pollution':ab,ti OR 'Air Pollutions':ab,ti OR 'Pollution, Air':ab,ti OR 'Air Quality':ab,ti OR 'Air Pollutants':ab,ti OR 'Air Pollutant':ab,ti OR 'Air Environmental Pollutants':ab,ti OR 'Environmental Air Pollutants':ab,ti OR 'Nitrogen Dioxide':ab,ti OR 'Dioxide, Nitrogen':ab,ti OR 'Nitrogen Peroxide':ab,ti OR 'Peroxide, Nitrogen':ab,ti OR 'Sulfur Dioxide':ab,ti OR 'Sulfurous Anhydride':ab,ti OR 'Carbon Monoxide':ab,ti OR 'Monoxide, Carbon':ab,ti OR 'Ozone':ab,ti OR 'Tropospheric Ozone':ab,ti OR 'Low Level Ozone':ab,ti OR 'Ground Level Ozone':ab,ti OR 'Particulate Matter':ab,ti OR 'Ultrafine Fibers':ab,ti OR 'Ultrafine Fiber':ab,ti OR 'Airborne Particulate Matter':ab,ti OR 'Particulate Air Pollutants':ab,ti OR 'Nitrogen Oxides':ab,ti OR 'Oxides, Nitrogen':ab,ti OR 'Nitrogen Oxide':ab,ti OR 'Oxide, Nitrogen':ab,ti OR 'PM2.5':ab,ti OR 'PM10':ab,ti OR 'NO2':ab,ti OR 'NOx':ab,ti OR 'O3':ab,ti OR 'SO2':ab,ti OR 'black carbon':ab,ti) AND ('infertility therapy'/exp OR 'in vitro fertilization'/exp OR 'intrauterine insemination'/exp OR 'assisted reproduction technique':ab,ti OR 'assisted reproduction techniques':ab,ti OR 'assisted reproduction technologies':ab,ti OR 'assisted reproduction technology':ab,ti OR 'assisted reproduction therapy':ab,ti OR 'assisted reproduction treatment;':ab,ti OR 'assisted reproductive technique':ab,ti OR 'assisted reproductive techniques':ab,ti OR 'assisted reproductive technology':ab,ti OR 'assisted reproductive therapy':ab,ti OR 'assisted reproductive treatment':ab,ti OR 'fertility therapy':ab,ti OR 'fertility treatment':ab,ti OR 'reproductive techniques, assisted':ab,ti OR 'extracorporeal fertilization':ab,ti OR 'fertilization in vitro':ab,ti OR 'in vitro fertilisation':ab,ti OR 'ivf (in vitro fertilization)':ab,ti OR 'testtube baby':ab,ti OR 'insemination, intrauterine':ab,ti OR 'uterine insemination':ab,ti) AND ('live birth'/exp OR 'live birth rate'/exp OR 'clinical pregnancy'/exp OR 'clinical pregnancy rate'/exp OR 'pregnancy'/exp OR 'oocyte quality'/exp OR 'ovarian response'/exp OR 'ovulation induction'/exp OR 'Live Births':ab,ti OR ' liveborn child':ab,ti OR 'liveborn progeny':ab,ti OR 'live birth rate':ab,ti OR 'Pregnancy Rate':ab,ti OR 'Pregnancy Rates':ab,ti OR ' Live-Birth Pregnancy Rates':ab,ti OR ' Live Birth Pregnancy Rates':ab,ti OR 'Live-Birth Pregnancy Rate':ab,ti OR 'Live Birth Pregnancy Rate':ab,ti OR 'egg quality':ti,ab OR 'quality of oocyte':ab,ti OR 'Ovocytes quality':ab,ti OR 'Ovocyte quality':ab,ti OR 'Ovarian Stimulation':ab,ti OR 'Ovarian Stimulations':ab,ti OR 'poor responders':ab,ti OR 'ovarian response':ab,ti OR 'Biochemical pregnancy':ab,ti OR 'Uterine pregnancys':ab,ti)

Filter Language = English

Filter Dates = none

*Results = 171*
