# Supplementary material for: Association between ambient air pollution exposure and pregnancy outcomes in women treated with assisted reproductive technology: an updated systematic review and meta-analysis
Source: BMC Public Health. 2025 May 2;25:1639. doi: 10.1186/s12889-024-19301-3 (PMC12046897; doi:10.1186/s12889-024-19301-3)
Supplement: Supplementary file 1 — Supplementary Material 1: Appendix 1. Preferred Reporting Items for Systematic reviews and Meta-Analysis (PRISMA) 2009 Checklist; Appendix 2. Details for the search strategy used within each database; Appendix 3. OHAT Risk of Bias Rating Tool for Human and Animal Studies; Appendix 4. Approach to assessing the certainty of evidence from systematic reviews; Appendix 5. (Table. S2. The details the ART methodology; Table. S2. Risk of bias assessment using the National Toxicology Program's Office of Health Assessment and Translation (NTP/OHAT) tiered risk of bias approach; Table. S3. Confidence rating: assessment of body evidence; Fig. S1. Funnel plot of publication bias in reported associations between exposure to ambient air pollution and clinical pregnancy; Fig. S2. Funnel plot of publication bias in reported associations between exposure to ambient air pollution and biochemical pregnancy; Fig. S3. Funnel plot of publication bias in reported associations between exposure to ambient air pollution and live birth). Appendix 6. Sensitivity analyses of the association between ambient air pollution exposure and pregnancy outcomes in women treated with assisted reproductive technologies. [file 12889_2024_19301_MOESM1_ESM.zip › Appendix 4.docx]

Appendix 3. Rating the body of evidence

We will apply the NTP/OHAT framework, based on the GRADE guidelines, to rate the confidence with the body of evidence, translate to a level of evidence and integrate the different streams of evidence to deliver the hazard identification conclusions (OHAT 2015). Accordingly, the quality and level of evidence will be evaluated, establishing an initial confidence rate and using a sequential process considering those factors that may affect (upgrading or downgrading) the confidence including the risk of bias, imprecision, publication bias, indirectness, magnitude, dose-response and plausible confounding. The rating process will be completed considering those upgrading and downgrading factors and balanced together to deliver a final rate. The final confidence will be translated to level of evidence.

**Initial rating of confidence**

The initial confidence rating will be determined by the main features determined by the study design:

1. The exposure to the substance is experimentally controlled
2. The exposure assessment demonstrates that exposures occurred prior to the development of the outcome (or concurrent with aggravation/amplification of an existing condition)
3. The outcome is assessed on the individual level (i.e., not through population aggregate data)
4. An appropriate comparison group is included in the study

Table S3. Relationship of confidence features and the main study designs (OHAT 2015).

| **Study Design** | **Controlled Exposure** | **Exposure Prior to Outcome** | **Individual Outcome Data** | **Comparison Group Used** | **Initial**  **Confidence**  **Rating** |
| --- | --- | --- | --- | --- | --- |
| **Huma**n **controlled trial** | likely | likely | likely | likely | **high** |
| **Cohort** | unlikely | may or may not | likely | likely | **low to moderate** |
| **Case-control** | unlikely | may or may not | likely | likely | **low to moderate** |
| **Cross-sectional** | unlikely | unlikely | likely | likely | **low** |

**Factors downgrading the confidence**

**Risk of bias**

The summary tables of risk of bias for each stream of evidence will analyzed in order to analyze the overall consistency, direction, magnitude and sources of bias. Downgrading for risk of bias should reflect the entire body of studies; therefore, the decision to downgrade should be applied conservatively. The decision to downgrade should be reserved for cases for which there is substantial risk of bias across most of the studies composing the body of evidence.

The NTP/OHAT’s risk of bias tiered approach considers some key elements or risk of bias domains of higher relevance to establish the classification criteria for each individual study. For observational human studies the key elements would typically include exposure assessment, outcome assessment, and confounding/selection.

Tier 1: A study must be rated as “definitely low” or “probably low” risk of bias for key elements AND have most other applicable items answered “definitely low” or “probably low” risk of bias.

Tier 2: Study meets neither the criteria for tiers.

Tier 3: A study must be rated as “definitely high” or “probably high” risk of bias for key elements AND have most other applicable items answered “definitely high” or “probably high” risk of bias.

The NTP/OHAT guidelines states: “*The strategy for assessing risk of bias differs depending on whether confidence ratings will be primarily used to identify research needs for a state-of-science evaluation or to reach formal NTP conclusions on hazard identification. Downgrading for risk of bias should reflect the entire body of studies; therefore, the decision to downgrade should be applied conservatively. The decision to downgrade should be reserved for cases for which there is substantial risk of bias across most of the studies composing the body of evidence*.”

Table S4. Criteria to rate the risk of bias. To downgrade the confidence will integrate the risk of bias from each study providing relevant information for the health outcomes of interest.

| “Not likely” | Most information is from Tier 1 studies (low risk of bias for all key domains).  Plausible bias unlikely to seriously alter the results |
| --- | --- |
| “Serious” | Most information is from Tier 1 and 2 studies.  Plausible bias that raises some doubt about the results |
| “Very serious” | The proportion of information from Tier 3 studies at high risk of bias for all key domains is sufficient to affect the interpretation of results.  Plausible bias that seriously weakens confidence in the results. |

**Imprecision**

The assessment of the 95% confidence intervals is the primary method to assess imprecision by NTP/OHAT in agreement with the GRADE approach (Guyatt, Oxman, Kunz, Brozek, et al. 2011).

Table S5. Criteria to rate the imprecision.

| Not serious | No or minimal indications of large standard deviations (i.e., SD > mean)  For ratio measures (e.g., odds ratio, OR) the ratio of the upper to lower 95% CI for most studies (or meta-estimate) is < 10; or for absolute measures (e.g., percent control response) the absolute difference between the upper and lower 95% CI for most studies (or meta-estimate) is < 100 |
| --- | --- |
| Serious | Does not clearly meet guidance for “not serious” or “very serious” |
| Very serious | Large standard deviations (i.e., SD > mean)  For ratio measures (e.g., OR) the ratio of the upper to lower 95% CI for most studies (or meta-estimate) is ≥ 10; or for absolute measures (e.g., percent control response) the absolute difference between the upper and lower 95% CI for most studies (or meta-estimate) is ≥ 100 |

For continuous variables GRADE guidelines states that review authors should consider downrating for imprecision whenever there are sample sizes lower than 400. Similar to the procedure for dichotomous variables, it is possible to calculate the optimal information size (OIS) setting the α and β error (suggested at 0.05 and 0.2, respectively), mean difference (Δ) and selecting an appropriate standard deviation. On that basis, using the usual standards of α (0.05) and β (0.20), and an effect size of 0.2 standard deviations, representing a small effect, requires a total sample size of approximately 400 (200 per group) a sample size that may not be sufficient to ensure prognostic balance (Guyatt, Oxman, Kunz, Brozek, et al. 2011).

**Publication bias**

The publication bias is defined by the “publication or non-publication of research findings, depending on the nature and direction of the results” (Higgins and Green 2011) and is assessed on the body of evidence, while the “selective outcome reporting” is assessed for each individual study during the risk of bias process (Guyatt, Oxman, Montori, et al. 2011). Downgrading by publication bias is only considered when the concern to reduce the confidence is serious (OHAT 2015).

We considered the issues outlined by NTP/OHAT in agreement with GRADE to rate the publication bias:

- Early positive studies, particularly if small in size, are suspect.
- Publication bias should be suspected when studies are uniformly small, particularly when sponsored by industries, non-government organizations, or authors with conflicts of interest.
- Funnel plots, Egger’s regression, and trim and fill techniques can be used to visualize asymmetrical or symmetrical patterns of study results to help assess publication bias when adequate data for a specific outcome are available. Funnel plots and other approaches are less reliable when there are only a few studies.
- The identification of abstracts or other types of grey literature that do not appear as full-length articles within a reasonable time frame (around 3 to 4 years) can be another indication of publication bias.

**Indirectness and applicability**

To assess the extent of the directness and applicability, NTP/OHAT approach considers (1) relevance of the animal model to outcome of concern (2) directness of the endpoints to the primary health outcome(s) (3) nature of the exposure in human studies and route of administration in animal studies (4) duration of treatment in animal studies and length of time between exposure and outcome assessment in animal and prospective human studies. Similarly, GRADE group identifies four types of indirectness: differences in population (applicability), differences in interventions (applicability), differences in outcome measures (surrogate outcomes) and indirect comparisons (Guyatt, Oxman, Kunz, Woodcock, Brozek, Helfand, Alonso-Coello, Falck-Ytter, et al. 2011).

We outlined the following points to assess the directness in the present study:

- Differences in population (applicability) and relevance of the animal model to outcome of concern

Human studies. We may rate down for population differences if there is a compelling reason to justify the biology in the population of interest is so different of the population assessed and thus, the magnitude may differ substantially.

- Differences in outcome measures (surrogate outcomes) or directness of the endpoints to the primary health outcome(s).

The applicability of specific health outcomes or biological processes in non-human animal models is outlined in the PECO-based inclusion and exclusion criteria, with the most accepted relevant/interpretable outcomes considered “primary” and less direct measures, biomarkers of effect, or upstream measures of health outcome considered “secondary”.

- Nature of the exposure in human studies and route of administration in animal studies (OHAT 2015).

Human studies. Human studies are not downgraded for directness regardless of the exposure level or setting (e.g., general population, occupational settings, etc.). In NTP/OHAT’s process, the applicability of a given exposure scenario for reaching a “level of concern” for a certain subpopulation is considered after hazard identification.

Dose levels used in animal studies: There is no downgrading for dose level used in experimental animal studies because it is not considered as a factor under directness for the purposes of reaching confidence ratings for evidence of health effects. NTP/OHAT recognizes that the level of dose or exposure is an important factor when considering the relevance of study findings. In NTP/OHAT’s process, consideration of dose occurs after hazard identification as part of reaching a “level of concern” conclusion when the health effect is interpreted in the context of what is known regarding the extent and nature of human exposure.

Route of administration in animal studies: External dose comparisons used to reach level of concern conclusions need to consider internal dosimetry in animal models, which can vary based on route of administration, species, age, diet, and other cofactors. The most commonly used routes of administration (i.e., oral, dermal, inhalation, subcutaneous) are generally considered direct for the purposes of establishing confidence ratings.

**Unexplained Inconsistency**

GRADE suggests rating down the quality of evidence if large inconsistency (heterogeneity) in study results remains after exploration of a priori hypotheses that might explain heterogeneity. Judgment of the extent of heterogeneity is based on similarity of point estimates, extent of overlap of confidence intervals, and statistical criteria including tests of heterogeneity and I^2^. Apparent subgroup effects should be interpreted cautiously with attention to whether subgroup comparisons come from within rather than between studies; if tests of interaction generate low P-values; and whether subgroup effects are based on a small number of a priori hypotheses with a specified direction (Guyatt, Oxman, Kunz, Woodcock, Brozek, Helfand, Alonso-Coello, Glasziou, et al. 2011). Inconsistency that can be explained, such as variability in study populations, would not be eligible for a downgrade. Potential sources of inconsistency across studies are explored, including consideration of population or animal model (e.g., cohort, species, strain, sex, life-stage at exposure and assessment); exposure or treatment duration, level, or timing relative to outcome; study methodology (e.g., route of administration, methodology used to measure health outcome); conflict of interest, and statistical power and risk of bias. Generally, there is no downgrade when identified sources of inconsistency can be attributed to study design features such as differences in species, timing of exposure, or health outcome assessment. There is no downgrade for inconsistency in cases where the evidence base consists of a single study. In this case, consistency is unknown and is documented as such in the summary of findings table (OHAT 2015).

A useful statistic for quantifying inconsistency is

$$I^{2}=\left( \frac{Q-df}{Q} \right) x 100 \%$$

where Q is the chi-squared statistic and df is its degrees of. This describes the percentage of the variability in effect estimates that is due to heterogeneity rather than sampling error (chance) (Higgins and Green 2011).

Thresholds for the interpretation of I^2^ can be misleading, since the importance of inconsistency depends on several factors. A rough guide to interpretation is as follows:

- 0% to 40%: might not be important;
- 30% to 60%: may represent moderate heterogeneity*;
- 50% to 90%: may represent substantial heterogeneity*;
- 75% to 100%: considerable heterogeneity*.

*The importance of the observed value of I^2^ depends on (i) magnitude and direction of effects and (ii) strength of evidence for heterogeneity (e.g. P value from the chi-squared test, or a confidence interval for I^2^) (Higgins and Green 2011).

Tau square (T^2^, tau^2^, τ^2^): An estimate of the between-study variance in a random-effects meta-analysis. A τ^2^ close to 0 would be strict homogeneity, and > 1 suggests the presence of substantial statistical heterogeneity (Higgins and Green 2011).

Table S6. Criteria to assess the inconsistency

| “Not serious” | Point estimates similar  Confidence intervals overlap  Statistical heterogeneity is non-significant (p ≥ 0.1)  I^2^ of ≤ 50% |
| --- | --- |
| “Serious” | Point estimates vary  Confidence intervals show minimal overlap  Statistical heterogeneity has low p-value (p ≤ 0.1)  I^2^ of > 50% to 75% |
| “Very serious” | Point estimates vary widely  Confidence intervals show minimal or no overlap  Statistical heterogeneity has low p-value (p ≤ 0.1)  I^2^ of > 75% |

**Factors upgrading the confidence**

We considered three factors to upgrade the confidence with the main bodies of evidence as stated by GRADE: magnitude of effect, dose-response/gradient and plausible confounders (Guyatt, Oxman, Sultan, et al. 2011).

**Magnitude**

Large magnitude of effect will be considered to upgrade the confidence on the basis of NTP/OHAT and GRADE guidance. Large magnitude in human studies is based primarily on modeling studies that suggest confounding alone is unlikely to explain associations with a relative risk (RR) greater than 2 (or less than 0.5) and very unlikely to explain associations with an RR greater than 5 (or less than 0.2) (Guyatt, Oxman, Sultan, et al. 2011; OHAT 2015)

**Dose-response**

We considered upgrading the dose-response if there is enough evidence of monotonic and non-monotonic gradient (OHAT 2015).

**Plausible confounding**

Sources of potential plausible confounding, also known as “residual confounding” or “residual bias” in epidemiology need to be investigated specially among the human body of evidence based with observational studies.

**Consistency**

The consistency is outlined by the NTP/OHAT protocol as upgrading factor considering the consistency across animal studies, dissimilar populations and study types.

Types of consistency according NTP/OHAT approach (OHAT 2015):

- *“across animal studies–consistent results reported in multiple experimental animal models or species”. There is no absolute definition of ‘consistency’ however finding the same direction of change in the same outcome in over two species would constitute sufficient evidence that a causal relationship has been established for IARC experimental evidence and consistency may be warranted (Preamble Part B Section 6).*
- *“across dissimilar populations–consistent results reported across populations (human or wildlife) that differ in factors such as time, location, and/or exposure “*
- *“across study types–consistent results reported from studies with different design features, e.g., between prospective cohort and case-control human studies or between chronic and multigenerational animal studies “*

**Final rate of confidence**

The final rate of confidence will be based on the judgement of all downgrading and upgrading factors over the initial rating. The final rates for each body of evidence are high confidence, moderate confidence and low confidence.

**Table S7. Summary for the confidence rating procedure**

| **Initial rating** | **Factors reducing confidence** | | | | | **Factors increasing confidence** | | | | **Final**  **rating** |
| --- | --- | --- | --- | --- | --- | --- | --- | --- | --- | --- |
|  | **Risk of bias** | **Unexplained inconsistency** | **Indirectness** | **Imprecision** | **Publication**  **bias** | **Magnitude** | **Dose**  **Response** | **Residual confounding** | **Consistency** |  |
| **High**  **Moderate**  **Low** | Unlikely/ Serious/Very serious | Serious /Not serious | Serious /Not serious | Serious /Not serious | Likely /Unlikely | Large /Not large | Large /Not large | Large /Not large | Large /Not large | **High**  **Moderate**  **Low** |

**Translation of confidence in the body of evidence into level of evidence for the health effect**

We used the descriptors proposed by NTP/OHAT to translate the level of confidence into the level of evidence for the health effect for each stream of evidence considering the confidence in the body of evidence and direction of the health effect.

Five descriptors are used by NTP/OHAT to defined the levels of evidence:

1. ***High level of evidence.*** *There is moderate confidence in the body of evidence for an association between exposure to the substance and the health outcome(s)*
2. ***Moderate level of evidence.*** *There is low confidence in the body of evidence for an association between exposure to the substance and the health outcome(s), or no data are available.*
3. ***Low level of evidence.*** *There is low confidence in the body of evidence for an association between exposure to the substance and the health outcome(s), or no data are available.*
4. ***Evidence of no health effect.*** *There is high confidence in the body of evidence that exposure to the substance is not associated with the health outcome(s).*
5. ***Inadequate evidence.*** *There is insufficient evidence available to assess if the exposure to the substance is associated with the health outcome(s).*

The direction or nature of the effect was considered in the translation process as following:

Table S8. Translation of confidence rating into level of evidence

| Confidence in the body of evidence | Direction of the effect  (Health effect) | Level of evidence for the health effect |
| --- | --- | --- |
| High | ⇨ | High |
| Moderate | ⇨ | Moderate |
| Low | ⇨ | Low |
| Very low or  no evidence | ⇨ | Inadequate |
| Confidence in the body of evidence | Direction of the effect  (No Health effect) | Level of evidence for the health effect |
| High | ⇨ | Evidence of no health effect |
| Moderate | ⇨ | Inadequate |
| Low | ⇨ | Inadequate |
| Very low or  no evidence | ⇨ | Inadequate |

REFERENCES

Guyatt GH, Oxman AD, Kunz R, Brozek J, Alonso-Coello P, Rind D, Devereaux PJ, Montori VM, Freyschuss B, Vist G, et al. GRADE guidelines 6. Rating the quality of evidence--imprecision. J Clin Epidemiol 2011: **64**; 1283-1293.

Guyatt GH, Oxman AD, Kunz R, Woodcock J, Brozek J, Helfand M, Alonso-Coello P, Falck-Ytter Y, Jaeschke R, Vist G, et al. GRADE guidelines: 8. Rating the quality of evidence--indirectness. J Clin Epidemiol 2011: **64**; 1303-1310.

Guyatt GH, Oxman AD, Kunz R, Woodcock J, Brozek J, Helfand M, Alonso-Coello P, Glasziou P, Jaeschke R, Akl EA, et al. GRADE guidelines: 7. Rating the quality of evidence--inconsistency. J Clin Epidemiol 2011: **64**; 1294-1302.

Guyatt GH, Oxman AD, Montori V, Vist G, Kunz R, Brozek J, Alonso-Coello P, Djulbegovic B, Atkins D, Falck-Ytter Y, et al. GRADE guidelines: 5. Rating the quality of evidence--publication bias. J Clin Epidemiol 2011: **64**; 1277-1282.

Guyatt GH, Oxman AD, Sultan S, Glasziou P, Akl EA, Alonso-Coello P, Atkins D, Kunz R, Brozek J, Montori V, et al. GRADE guidelines: 9. Rating up the quality of evidence. J Clin Epidemiol 2011: **64**; 1311-1316.

Higgins JPT and Green S. Cochrane Handbook for Systematic Reviews of Interventions Version 5.1.0. 2011. The Cochrane Collaboration.

OHAT. Handbook for Conducting a Literature-Based Health Assessment Using OHAT Approach for Systematic Review and Evidence Integration. In Office of Health Assessment and Translation (OHAT), DotNTP (ed). 2015. National Institute of Environmental Health Sciences.

OHAT. OHAT Risk of Bias Rating Tool for Human and Animal Studies. In Office of Health Assessment and Translation (OHAT), DotNTP (ed). 2015. National Institute of Environmental Health Science.
