# Supplementary material for: Association between ambient air pollution exposure and pregnancy outcomes in women treated with assisted reproductive technology: an updated systematic review and meta-analysis
Source: BMC Public Health. 2025 May 2;25:1639. doi: 10.1186/s12889-024-19301-3 (PMC12046897; doi:10.1186/s12889-024-19301-3)

**Table. S1.** The details the ART methodology

| **Stage** | **Description** | **Key Factors** |
| --- | --- | --- |
| Ovarian Stimulation | Physicians select stimulation protocols based on patient factors such as age, ovarian reserve markers (AMH, FSH, AFC), and BMI. Protocol options include: Long GnRH-a (agonist), Short GnRH-a (agonist), GnRH antagonist, or mild stimulation protocols. Duration typically spans 8 to 14 days. | Protocol selection based on patient profile;  Monitoring of ovarian response through ultrasound and hormone levels |
| Oocyte Retrieval | Mature oocytes are retrieved transvaginally under ultrasound guidance using a needle aspiration technique. Follicular flushing may be performed to maximize oocyte yield. Oocytes are evaluated for maturity and quality in the laboratory. | Transvaginal retrieval under ultrasound guidance;  Follicular flushing if needed;  Assessment of oocyte quality and maturity |
| Embryo Culture | Retrieved oocytes are cultured in a controlled environment until they reach the blastocyst stage. Embryos are assessed for morphology, developmental stage, and quality. Culture conditions may include varying media compositions and gas concentrations to optimize embryo development. | Culture to blastocyst stage;  Morphological and developmental assessment;  Optimization of culture conditions |
| Embryo Transfer | Suitable embryos are selected based on morphology, developmental stage, and viability. Transfer timing may be based on embryo quality and patient factors. The number of embryos transferred is determined based on patient age, embryo quality, and previous ART outcomes. Catheter selection and transfer technique are crucial for successful implantation. | Selection criteria for embryos;  Transfer timing and number of embryos;  Catheter type and transfer technique |
| Luteal Phase Support | Progesterone supplementation is provided post-embryo transfer to support endometrial receptivity and implantation. Administration routes include vaginal, intramuscular, or oral. Monitoring of serum progesterone levels may be performed to ensure adequate supplementation. | Progesterone supplementation post-transfer;  Monitoring of progesterone levels |
| Pregnancy Testing | Blood tests are conducted 9-11 days post-embryo transfer to measure levels of hCG, a hormone produced by the developing embryo. Positive results indicate pregnancy, and further monitoring is initiated to assess viability and ongoing pregnancy progression. | Blood test for hCG levels |

**Table. S2.** Risk of bias assessment using the National Toxicology Program's Office of Health Assessment and Translation (NTP/OHAT) tiered risk of bias approach.

| Studies | Selection bias | Confounding bias | Attrition/Exclusion bias | Exposure characterization | Outcome assessment | Selective reporting bias | Conflict of interest | Summary tiered classification |
| --- | --- | --- | --- | --- | --- | --- | --- | --- |
| Boulet et al. 2019 | PL | PH | PL | PL | PL | PL | PL | Tier 2 |
| Choe et al. 2018 | PL | PH | PL | PH | PL | PL | PL | Tier 2 |
| Dai et al. 2021 | PL | PL | PL | PH | PL | PL | PL | Tier 2 |
| Gonzalez-Comadran et al. 2021 | PL | PL | PL | PL | PL | PL | PL | Tier 1 |
| Iodice et al. 2021 | PL | PH | PL | PL | PL | PL | PL | Tier 2 |
| Jin et al. 2022 | PL | PH | PL | PH | PL | PL | PL | Tier 2 |
| Legro et al. 2010 | PL | PH | PL | PL | PL | PL | PL | Tier 2 |
| Li et al. 2020 | PL | PL | PL | PL | PL | PL | PL | Tier 1 |
| Liu et al. 2022 | PL | PH | PL | PH | PL | PL | PL | Tier 2 |
| Perin et al. 2010 | PL | PH | PL | PH | PL | PL | PL | Tier 2 |
| Qiu et al. 2019 | PL | PL | PL | PH | PL | PL | PL | Tier 2 |
| Quraishi et al. 2019 | PL | PH | PL | PL | PL | PL | PL | Tier 2 |
| Shi et al. 2021 | PL | PL | PL | PH | PL | PL | PL | Tier 2 |
| Tartaglia et al. 2022 | PL | PH | PL | PH | PL | PL | PL | Tier 2 |
| Wan et al. 2022 | PL | PH | PL | PH | PL | PL | PL | Tier 2 |
| Wang et al. 2019 | PL | PL | PL | PH | PL | PL | PL | Tier 2 |
| Wang et al. 2023 | PL | PL | PL | PL | PL | PL | PL | Tier 2 |
| Wu et al. 2021 | PL | PL | PL | PH | PL | PL | PL | Tier 2 |
| Zeng et al. 2020 | PL | PH | PL | PH | PL | PL | PL | Tier 2 |
| Zhang et al. 2022 | PL | PL | PL | PH | PL | PL | PL | Tier 2 |

Notes: DL, definitely low; PL, probably low; PH, probably high; DH, definitely high.

Tier 1: A study must be rated as “definitely low” or “probably low” risk of bias for key elements AND have most other applicable items rated “definitely low” or “probably low” risk of bias.

Tier 2: Study meets neither criterion for 1st or 3rd tiers.

Tier 3: A study must be rated as “definitely high” or “probably high” risk of bias for key elements AND have most other applicable items rated “definitely high” or “probably high risk of bias.

GRADE Evidence profile.

**Table. S3.** Confidence rating: assessment of body evidence.

| Air pollutant | Exposure period | Initial rate of confidence | Downgrading factors | | | | | Upgrading factors | | | | Certainty of the evidence (GRADE) |
| --- | --- | --- | --- | --- | --- | --- | --- | --- | --- | --- | --- | --- |
|  |  |  | Risk of bias | Inconsistency | Indirectness | Imprecision | Publication bias | Large magnitude  of association | Dose response | Residual confounding | Consistency |  |
| **Clinical pregnancy** | | | | | | | | | | | | |
| **CO** | Any period of assisted reproductive process | Moderate | Serious | Serious | Not likely | Not likely | Serious | No upgrade | No upgrade | No upgrade | No upgrade | **Low** |
|  | Ovarian stimulation to oocyte retrieval | Moderate | Serious | Serious | Not likely | Not likely | Not likely | No upgrade | No upgrade | No upgrade | No upgrade | **Moderate** |
|  | Oocyte retrieval to embryo transfer | Moderate | Serious | Not likely | Not likely | Not likely | Not likely | No upgrade | No upgrade | No upgrade | No upgrade | **Moderate** |
|  | Embryo transfer to hCG test | Moderate | Serious | Very Serious | Not likely | Not likely | Not likely | No upgrade | No upgrade | No upgrade | No upgrade | **Low** |
| **NO_2_** | Any period of assisted reproductive process | Moderate | Serious | Serious | Not likely | Not likely | Not likely | No upgrade | No upgrade | No upgrade | No upgrade | **Moderate** |
|  | Ovarian stimulation to oocyte retrieval | Moderate | Serious | Not likely | Not likely | Not likely | Not likely | No upgrade | No upgrade | No upgrade | No upgrade | **Moderate** |
|  | Oocyte retrieval to embryo transfer | Moderate | Serious | Not likely | Not likely | Not likely | Not likely | No upgrade | No upgrade | No upgrade | No upgrade | **Moderate** |
|  | Embryo transfer to hCG test | Moderate | Serious | Serious | Not likely | Not likely | Not likely | No upgrade | No upgrade | No upgrade | No upgrade | **Moderate** |
| **O_3_** | Any period of assisted reproductive process | Moderate | Serious | Very Serious | Not likely | Not likely | Not likely | No upgrade | No upgrade | No upgrade | No upgrade | **Low** |
|  | Ovarian stimulation to oocyte retrieval | Moderate | Serious | Very Serious | Not likely | Not likely | Not likely | No upgrade | No upgrade | No upgrade | No upgrade | **Low** |
|  | Oocyte retrieval to embryo transfer | Moderate | Serious | Very Serious | Not likely | Not likely | Not likely | No upgrade | No upgrade | No upgrade | No upgrade | **Low** |
|  | Embryo transfer to hCG test | Moderate | Serious | Very Serious | Not likely | Not likely | Not likely | No upgrade | No upgrade | No upgrade | No upgrade | **Low** |
| **PM_10_** | Any period of assisted reproductive process | Moderate | Serious | Serious | Not likely | Not likely | Not likely | No upgrade | No upgrade | No upgrade | No upgrade | **Moderate** |
|  | Ovarian stimulation to oocyte retrieval | Moderate | Serious | Not likely | Not likely | Not likely | Not likely | No upgrade | No upgrade | No upgrade | No upgrade | **Moderate** |
|  | Oocyte retrieval to embryo transfer | Moderate | Serious | Not likely | Not likely | Not likely | Not likely | No upgrade | No upgrade | No upgrade | No upgrade | **Moderate** |
|  | Embryo transfer to hCG test | Moderate | Serious | Serious | Not likely | Not likely | Not likely | No upgrade | No upgrade | No upgrade | No upgrade | **Moderate** |
| **PM_2.5_** | Any period of assisted reproductive process | Moderate | Serious | Serious | Not likely | Not likely | Not likely | No upgrade | No upgrade | No upgrade | No upgrade | **Moderate** |
|  | Ovarian stimulation to oocyte retrieval | Moderate | Serious | Serious | Not likely | Not likely | Not likely | No upgrade | No upgrade | No upgrade | No upgrade | **Moderate** |
|  | Oocyte retrieval to embryo transfer | Moderate | Serious | Not likely | Not likely | Not likely | Not likely | No upgrade | No upgrade | No upgrade | No upgrade | **Moderate** |
|  | Embryo transfer to hCG test | Moderate | Serious | very Serious | Not likely | Not likely | Not likely | No upgrade | No upgrade | No upgrade | No upgrade | **Low** |
| **SO_2_** | Any period of assisted reproductive process | Moderate | Serious | Serious | Not likely | Not likely | Serious | No upgrade | No upgrade | No upgrade | No upgrade | **Moderate** |
|  | Ovarian stimulation to oocyte retrieval | Moderate | Serious | Serious | Not likely | Not likely | Not likely | No upgrade | No upgrade | No upgrade | No upgrade | **Moderate** |
|  | Oocyte retrieval to embryo transfer | Moderate | Serious | very Serious | Not likely | Not likely | Not likely | No upgrade | No upgrade | No upgrade | No upgrade | **Low** |
|  | Embryo transfer to hCG test | Moderate | Serious | very Serious | Not likely | Not likely | Not likely | No upgrade | No upgrade | No upgrade | No upgrade | **Low** |
| **Biochemical pregnancy** | | | | | | | | | | | | |
| **CO** | Any period of assisted reproductive process | Moderate | Serious | Not likely | Not likely | Not likely | Not likely | No upgrade | No upgrade | No upgrade | No upgrade | **Moderate** |
|  | Ovarian stimulation to oocyte retrieval | Moderate | Serious | Not likely | Not likely | Not likely | Not likely | No upgrade | No upgrade | No upgrade | No upgrade | **Moderate** |
|  | Oocyte retrieval to embryo transfer | Moderate | Serious | Not likely | Not likely | Not likely | Not likely | No upgrade | No upgrade | No upgrade | No upgrade | **Moderate** |
|  | Embryo transfer to hCG test | Moderate | Serious | Not likely | Not likely | Not likely | Not likely | No upgrade | No upgrade | No upgrade | No upgrade | **Moderate** |
| **NO_2_** | Any period of assisted reproductive process | Moderate | Serious | Serious | Not likely | Not likely | Not likely | No upgrade | No upgrade | No upgrade | No upgrade | **Moderate** |
|  | Ovarian stimulation to oocyte retrieval | Moderate | Serious | Not likely | Not likely | Not likely | Not likely | No upgrade | No upgrade | No upgrade | No upgrade | **Moderate** |
|  | Oocyte retrieval to embryo transfer | Moderate | Serious | Serious | Not likely | Not likely | Not likely | No upgrade | No upgrade | No upgrade | No upgrade | **Moderate** |
|  | Embryo transfer to hCG test | Moderate | Serious | very Serious | Not likely | Not likely | Not likely | No upgrade | No upgrade | No upgrade | No upgrade | **Low** |
| **O_3_** | Any period of assisted reproductive process | Moderate | Serious | Serious | Not likely | Not likely | Serious | No upgrade | No upgrade | No upgrade | No upgrade | **Moderate** |
|  | Ovarian stimulation to oocyte retrieval | Moderate | Serious | Serious | Not likely | Not likely | Not likely | No upgrade | No upgrade | No upgrade | No upgrade | **Moderate** |
|  | Oocyte retrieval to embryo transfer | Moderate | Serious | Serious | Not likely | Not likely | Not likely | No upgrade | No upgrade | No upgrade | No upgrade | **Moderate** |
|  | Embryo transfer to hCG test | Moderate | Serious | Not likely | Not likely | Not likely | Not likely | No upgrade | No upgrade | No upgrade | No upgrade | **Moderate** |
| **PM_10_** | Any period of assisted reproductive process | Moderate | Serious | Serious | Not likely | Not likely | Not likely | No upgrade | No upgrade | No upgrade | No upgrade | **Moderate** |
|  | Ovarian stimulation to oocyte retrieval | Moderate | Serious | Serious | Not likely | Not likely | Not likely | No upgrade | No upgrade | No upgrade | No upgrade | **Moderate** |
|  | Oocyte retrieval to embryo transfer | Moderate | Serious | Serious | Not likely | Not likely | Not likely | No upgrade | No upgrade | No upgrade | No upgrade | **Moderate** |
|  | Embryo transfer to hCG test | Moderate | Serious | Very serious | Not likely | Not likely | Not likely | No upgrade | No upgrade | No upgrade | No upgrade | **Low** |
| **PM_2.5_** | Any period of assisted reproductive process | Moderate | Serious | Not likely | Not likely | Not likely | Serious | No upgrade | No upgrade | No upgrade | No upgrade | **Moderate** |
|  | Ovarian stimulation to oocyte retrieval | Moderate | Serious | Serious | Not likely | Not likely | Not likely | No upgrade | No upgrade | No upgrade | No upgrade | **Moderate** |
|  | Oocyte retrieval to embryo transfer | Moderate | Serious | Not likely | Not likely | Not likely | Not likely | No upgrade | No upgrade | No upgrade | No upgrade | **Moderate** |
|  | Embryo transfer to hCG test | Moderate | Serious | Serious | Not likely | Not likely | Not likely | No upgrade | No upgrade | No upgrade | No upgrade | **Moderate** |
| **SO_2_** | Any period of assisted reproductive process | Moderate | Serious | Serious | Not likely | Not likely | Serious | No upgrade | No upgrade | No upgrade | No upgrade | **Moderate** |
|  | Ovarian stimulation to oocyte retrieval | Moderate | Serious | Very serious | Not likely | Not likely | Not likely | No upgrade | No upgrade | No upgrade | No upgrade | **Low** |
|  | Oocyte retrieval to embryo transfer | Moderate | Serious | Very serious | Not likely | Not likely | Not likely | No upgrade | No upgrade | No upgrade | No upgrade | **Low** |
|  | Embryo transfer to hCG test | Moderate | Serious | Serious | Not likely | Not likely | Not likely | No upgrade | No upgrade | No upgrade | No upgrade | **Moderate** |
| **Live birth** | | | | | | | | | | | | |
| **CO** | Any period of assisted reproductive process | Moderate | Serious | Not likely | Not likely | Not likely | Not likely | No upgrade | No upgrade | No upgrade | No upgrade | **Moderate** |
|  | Ovarian stimulation to oocyte retrieval | Moderate | Serious | Not likely | Not likely | Not likely | Not likely | No upgrade | No upgrade | No upgrade | No upgrade | **Moderate** |
|  | Oocyte retrieval to embryo transfer | Moderate | Serious | Not likely | Not likely | Not likely | Not likely | No upgrade | No upgrade | No upgrade | No upgrade | **Moderate** |
|  | Embryo transfer to hCG test | Moderate | Serious | Not likely | Not likely | Not likely | Not likely | No upgrade | No upgrade | No upgrade | No upgrade | **Moderate** |
| **NO_2_** | Any period of assisted reproductive process | Moderate | Serious | Very serious | Not likely | Not likely | Serious | No upgrade | No upgrade | No upgrade | No upgrade | **Low** |
|  | Ovarian stimulation to oocyte retrieval | Moderate | Serious | Very serious | Not likely | Not likely | Not likely | No upgrade | No upgrade | No upgrade | No upgrade | **Low** |
|  | Oocyte retrieval to embryo transfer | Moderate | Serious | Serious | Not likely | Not likely | Not likely | No upgrade | No upgrade | No upgrade | No upgrade | **Moderate** |
|  | Embryo transfer to hCG test | Moderate | Serious | Very serious | Not likely | Not likely | Not likely | No upgrade | No upgrade | No upgrade | No upgrade | **Low** |
| **O_3_** | Any period of assisted reproductive process | Moderate | Serious | Very serious | Not likely | Not likely | Not likely | No upgrade | No upgrade | No upgrade | No upgrade | **Low** |
|  | Ovarian stimulation to oocyte retrieval | Moderate | Serious | Very serious | Not likely | Not likely | Not likely | No upgrade | No upgrade | No upgrade | No upgrade | **Low** |
|  | Oocyte retrieval to embryo transfer | Moderate | Serious | Serious | Not likely | Not likely | Not likely | No upgrade | No upgrade | No upgrade | No upgrade | **Moderate** |
|  | Embryo transfer to hCG test | Moderate | Serious | Very serious | Not likely | Not likely | Not likely | No upgrade | No upgrade | No upgrade | No upgrade | **Low** |
| **PM_10_** | Any period of assisted reproductive process | Moderate | Serious | Not likely | Not likely | Not likely | Not likely | No upgrade | No upgrade | No upgrade | No upgrade | **Moderate** |
|  | Ovarian stimulation to oocyte retrieval | Moderate | Serious | Serious | Not likely | Not likely | Serious | No upgrade | No upgrade | No upgrade | No upgrade | **Moderate** |
|  | Oocyte retrieval to embryo transfer | Moderate | Serious | Not likely | Not likely | Not likely | Not likely | No upgrade | No upgrade | No upgrade | No upgrade | **Moderate** |
|  | Embryo transfer to hCG test | Moderate | Serious | Not likely | Not likely | Not likely | Not likely | No upgrade | No upgrade | No upgrade | No upgrade | **Moderate** |
| **PM_2.5_** | Any period of assisted reproductive process | Moderate | Serious | Not likely | Not likely | Not likely | Not likely | No upgrade | No upgrade | No upgrade | No upgrade | **Moderate** |
|  | Ovarian stimulation to oocyte retrieval | Moderate | Serious | Not likely | Not likely | Not likely | Not likely | No upgrade | No upgrade | No upgrade | No upgrade | **Moderate** |
|  | Oocyte retrieval to embryo transfer | Moderate | Serious | Not likely | Not likely | Not likely | Not likely | No upgrade | No upgrade | No upgrade | No upgrade | **Moderate** |
|  | Embryo transfer to hCG test | Moderate | Serious | Not likely | Not likely | Not likely | Not likely | No upgrade | No upgrade | No upgrade | No upgrade | **Moderate** |
| **SO_2_** | Any period of assisted reproductive process | Moderate | Serious | Not likely | Not likely | Not likely | Serious | No upgrade | No upgrade | No upgrade | No upgrade | **Moderate** |
|  | Ovarian stimulation to oocyte retrieval | Moderate | Serious | Not likely | Not likely | Not likely | Not likely | No upgrade | No upgrade | No upgrade | No upgrade | **Moderate** |
|  | Oocyte retrieval to embryo transfer | Moderate | Serious | Serious | Not likely | Not likely | Not likely | No upgrade | No upgrade | No upgrade | No upgrade | **Moderate** |
|  | Embryo transfer to hCG test | Moderate | Serious | Not likely | Not likely | Not likely | Not likely | No upgrade | No upgrade | No upgrade | No upgrade | **Moderate** |

**Fig. S1.** Funnel plot of publication bias in reported associations between exposure to ambient air pollution and clinical pregnancy.


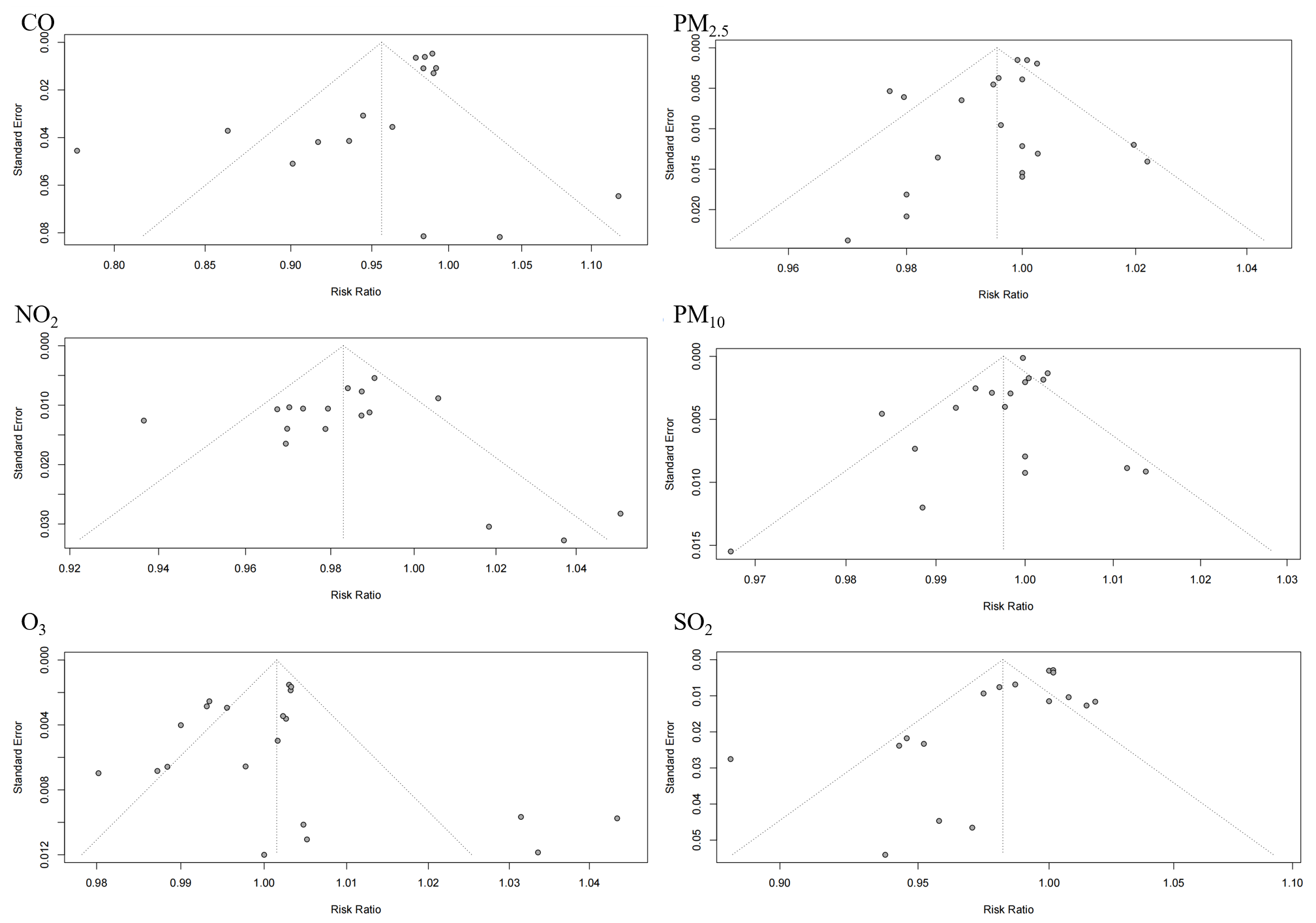


**Fig. S2.** Funnel plot of publication bias in reported associations between exposure to ambient air pollution and biochemical pregnancy.


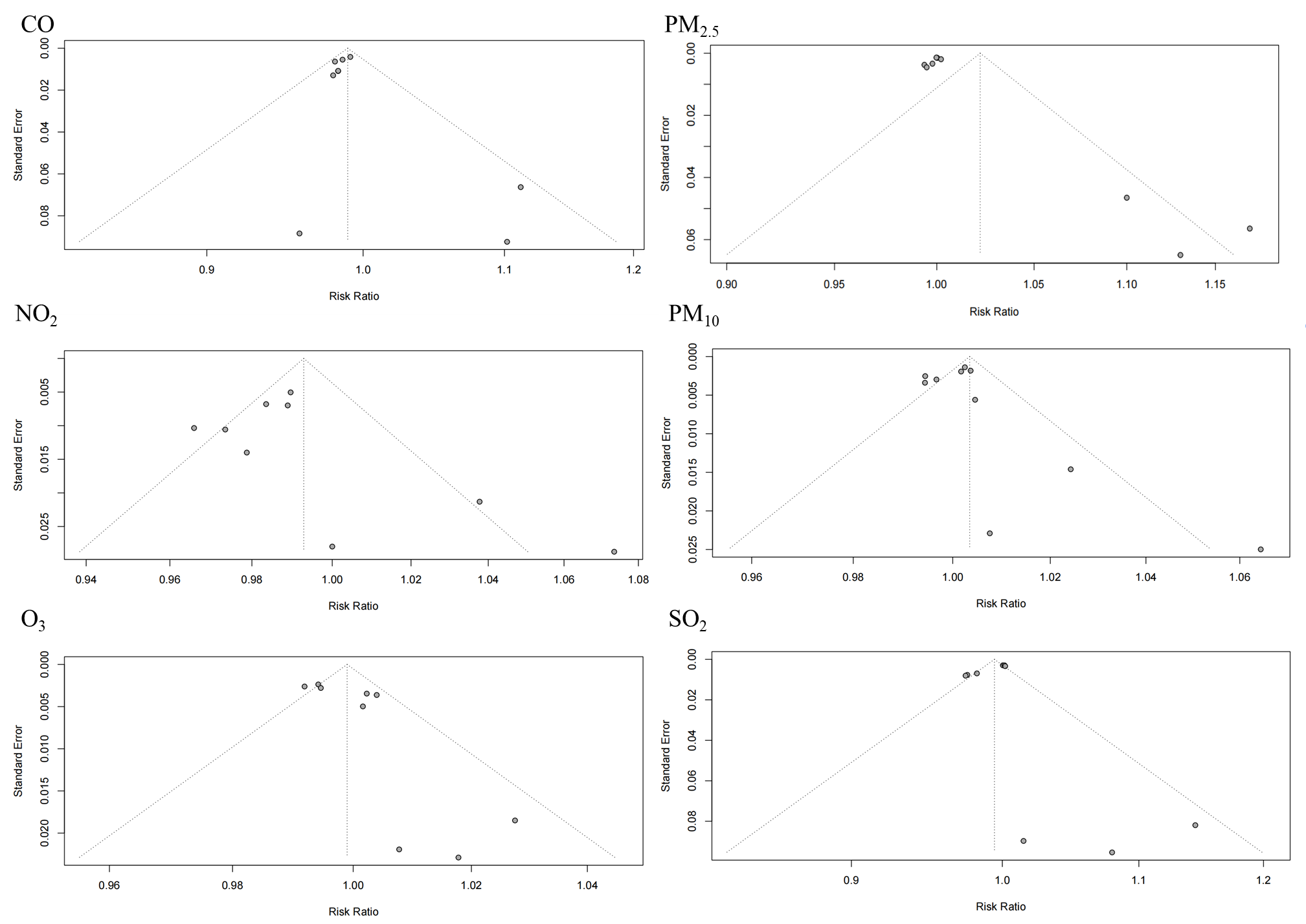


**Fig. S3.** Funnel plot of publication bias in reported associations between exposure to ambient air pollution and live birth.


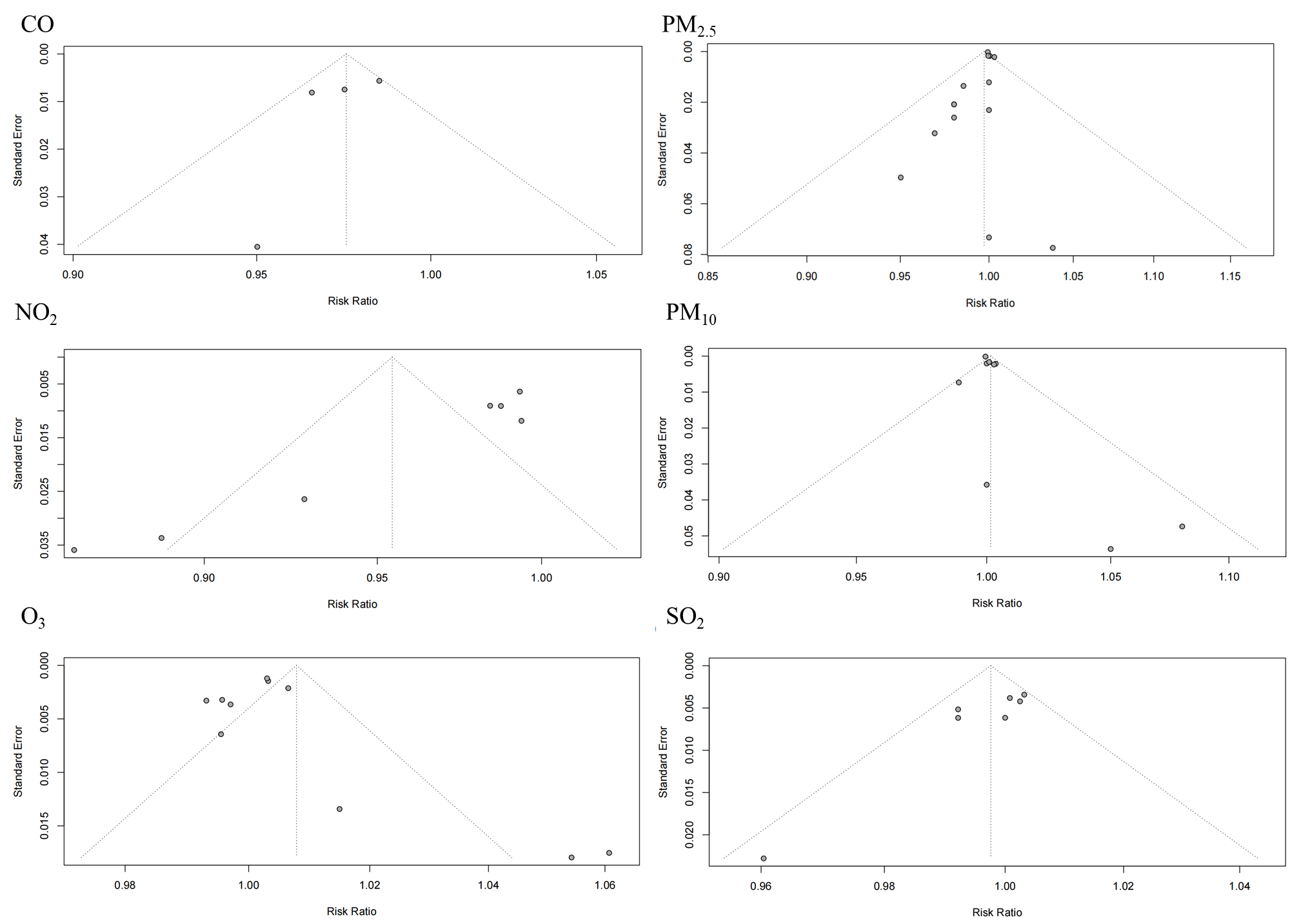

Supplement: Supplementary file 1 — Supplementary Material 1: Appendix 1. Preferred Reporting Items for Systematic reviews and Meta-Analysis (PRISMA) 2009 Checklist; Appendix 2. Details for the search strategy used within each database; Appendix 3. OHAT Risk of Bias Rating Tool for Human and Animal Studies; Appendix 4. Approach to assessing the certainty of evidence from systematic reviews; Appendix 5. (Table. S2. The details the ART methodology; Table. S2. Risk of bias assessment using the National Toxicology Program's Office of Health Assessment and Translation (NTP/OHAT) tiered risk of bias approach; Table. S3. Confidence rating: assessment of body evidence; Fig. S1. Funnel plot of publication bias in reported associations between exposure to ambient air pollution and clinical pregnancy; Fig. S2. Funnel plot of publication bias in reported associations between exposure to ambient air pollution and biochemical pregnancy; Fig. S3. Funnel plot of publication bias in reported associations between exposure to ambient air pollution and live birth). Appendix 6. Sensitivity analyses of the association between ambient air pollution exposure and pregnancy outcomes in women treated with assisted reproductive technologies. [file 12889_2024_19301_MOESM1_ESM.zip › Appendix 5.docx]
